# Non-Coding RNAs in Cancer: Decoding Regulatory Networks for Liquid Biopsy Applications

**DOI:** 10.3390/genes17040446

**Published:** 2026-04-13

**Authors:** Evelina Charidemou, Christos Papaneophytou

**Affiliations:** Department of Life Sciences, School of Life and Health Sciences, University of Nicosia, Nicosia 2417, Cyprus; charidemou.e@unic.ac.cy

**Keywords:** non-coding RNAs, circulating ncRNAs, multianalyte liquid biopsy, cancer attractor states, regulatory networks, biomarker panels, cell-free DNA, cancer biomarkers

## Abstract

Non-coding RNAs (ncRNAs) have emerged as important regulators of gene expression and cellular homeostasis, and their dysregulation is now recognized as a hallmark of cancer. Over the past decades, extensive research has demonstrated that diverse ncRNA classes, including microRNAs (miRNAs), long non-coding RNAs (lncRNAs), circular RNAs (circRNAs), and other small ncRNA species, participate in complex regulatory networks that influence tumor initiation, progression, metastasis, and therapy response. Through mechanisms such as transcriptional regulation, post-transcriptional gene silencing, epigenetic modulation, and competitive endogenous RNA interactions, ncRNAs shape the molecular circuitry underlying cancer development. In addition to their functional roles in tumor biology, many ncRNAs are released into biological fluids and can be detected as circulating molecules in blood, urine, saliva, and other biofluids. Their remarkable stability in extracellular environments has generated considerable interest in their use as minimally invasive biomarkers in liquid biopsy applications. Emerging evidence has shown that circulating ncRNAs (c-ncRNAs) can support cancer detection, disease stratification, and treatment monitoring. This narrative review provides an integrated view that links ncRNA-mediated regulatory networks with their application as liquid biopsy biomarkers, positioning ncRNAs as comprehensive indicators of tumor conditions. Particular emphasis is placed on c-ncRNA biomarkers, the integration of multiple ncRNA classes, and multi-analyte biomarker strategies that combine ncRNAs with complementary circulating molecules such as cell-free DNA and protein markers. Finally, we discuss the technical and clinical challenges that currently limit the translation of ncRNA-based diagnostics into clinical practice and highlight future directions for advancing ncRNA-guided liquid biopsy approaches in precision oncology.

## 1. Introduction

Cancer remains one of the biggest challenges to global public health. The Global Burden of Disease (GBD) 2023 study estimated 18.5 million new cancer cases (95% uncertainty interval [UI] 16.4–20.7 million; excluding non-melanoma skin cancer) and 10.4 million cancer-related deaths (95% UI 9.6–10.9 million) in 2023, confirming cancer as the second leading cause of death worldwide [1,2]. Breast cancer was the most commonly diagnosed cancer, followed by cancers of the trachea, bronchus, lung, colorectal cancer (CRC), prostate cancer, and stomach cancer. In contrast, lung cancer remained the leading cause of cancer death [1]. Notably, in 2023, 41.7% (95% UI 37.8–45.4%) of cancer deaths were linked to known behavioral and environmental risk factors [1]. The burden is projected to rise significantly, with an estimated 30.5 million (95% UI 22.9–38.9 million) new diagnoses and 18.6 million (95% UI 15.6–21.5 million) deaths expected each year by 2050, representing increases of 60.7% and 74.5%, respectively [1,2]. This remarkable diversity across organ systems, combined with extensive molecular heterogeneity within and between tumors, poses a significant challenge for early diagnosis, accurate prognosis, and effective treatment [3].

At the molecular level, cancer develops through the gradual buildup of genetic and epigenetic changes that disturb normal cellular balance [4,5,6]. These changes support the development of essential capabilities, including sustained proliferative signaling, evasion of growth suppressors, resistance to cell death, replicative immortality, induction of angiogenesis, and activation of invasion and metastasis programs [7]. The biological complexity of cancer is further increased by dynamic interactions within the tumor microenvironment, clonal evolution under selective pressures, and phenotypic plasticity that allows for adaptation to therapeutic interventions [8]. Recognizing that this complexity cannot be fully captured by single-gene or single-pathway analyses, the research community has increasingly adopted system-level approaches [9].

Large-scale collaborative initiatives, including The Cancer Genome Atlas (TCGA) [10,11] and the International Cancer Genome Consortium (ICGC) [12], have generated ex-tensive multi-omics datasets that encompass somatic mutations, copy number variations, DNA methylation profiles, transcriptomes, and proteomes across thousands of tumors representing diverse cancer types. These resources have significantly advanced our molecular understanding of tumor development and have enabled the systematic identification of dysregulated ncRNAs across different cancer types such as breast, lung and prostate cancers. For example, TCGA-based analyses have revealed recurrent miRNA alterations, including the overexpression of oncogenic miRNAs such as miR-21 and miR-155 in multiple malignancies, including breast, lung, CRC, gastric, hepatocellular, pancreatic, and prostate cancers [13,14,15]. Meanwhile, TCGA transcriptomic datasets demonstrate cancer type-specific and prognostically relevant long non-coding RNA (lncRNA) expression patterns, highlighting the broader non-coding RNA landscape that influences tumor phenotype [13,14]. Importantly, integrative analyses of these datasets have also facilitated the reconstruction of ncRNA-centered regulatory networks, including microRNA (miRNA)–mRNA interactions and competing endogenous RNA (ceRNA) networks, offering insights into how ncRNAs coordinate gene expression programs in cancer [13,14,15]. These findings emphasize the need for analytical frameworks that can capture the complex regulatory architecture of tumor systems. Notably, pan-cancer analyses have shown that tumors arising in different anatomical sites may share greater molecular similarities than tumors of the same histological type, further challenging traditional organ-based classification systems and supporting a shift toward molecularly driven diagnostic and therapeutic strategies [16].

Despite major advances in molecular characterization, routine clinical oncology still mainly depends on two primary methods for detecting and monitoring cancer: (i) invasive or costly diagnostic procedures, such as endoscopy, low-dose computed tomography, and tissue biopsy, with the latter remaining the gold standard for tumor diagnosis but requiring invasive tissue sampling; and (ii) minimally invasive or non-invasive approaches that analyze tumor-related components in body fluids, broadly called liquid biopsy, which enables dynamic, real-time monitoring of cancer [17].

In oncology, a liquid biopsy involves detecting circulating tumor-derived molecules in biological fluids such as blood, saliva, and urine, and is a promising method for cancer screening, diagnosis, and disease monitoring [18]. However, in current clinical practice, liquid biopsy-based assessment still relies on a relatively small set of circulating protein biomarkers, known as the “classical” tumor markers. These include, among others, Prostate-Specific Antigen (PSA) for prostate cancer, Cancer Antigen 125 (CA-125) for ovarian cancer, Carcinoembryonic Antigen (CEA) for CRC, Alpha-fetoprotein (AFP) for hepatocellular carcinoma, and Cancer Antigen 19-9 (CA 19-9) for pancreatic cancer.

Despite their widespread use, these biomarkers exhibit suboptimal diagnostic performance, particularly in early-stage disease. For example, PSA testing suffers from well-recognized limitations in diagnostic specificity. A recent systematic review of symptomatic men reported a PSA sensitivity of 0.93 but a specificity of only 0.20, indicating that up to 80% of men without cancer may test positive, thereby contributing to high false-positive rates and unnecessary biopsies [19]. Similarly, PSA performance is particularly poor within the “gray zone” of 4–10 ng/mL, where specificity declines sharply, leading to a substantial number of negative biopsies and overdiagnosis [20]. CA-125 also demonstrates limited sensitivity for early-stage ovarian cancer, detecting only approximately 50% of Stage I–II cases, compared with over 90% in advanced-stage disease [21]. Likewise, CA 19-9 has limited value for early pancreatic cancer detection, with reported sensitivities of only 40–50% in Stage I disease. Its specificity is further compromised by elevations in benign conditions such as pancreatitis, cholangitis, and obstructive jaundice, leading to clinically significant false-positive results [22]. These limitations have contributed to the unsuccessful implementation of several biomarker-based screening strategies in the general population, as the balance between sensitivity, specificity, and clinical benefit remains inadequate. For these reasons, although classical tumor markers retain value in disease monitoring and prognosis, their utility as standalone diagnostic tools is limited, highlighting the need for more accurate and integrative biomarker approaches (reviewed in [23]).

Importantly, early detection remains crucial for improving patient outcomes. For many cancer types (e.g., breast cancer), 5-year survival rates for early-stage disease can exceed 90%, whereas late-stage detection is often associated with markedly poorer outcomes, with survival in some cancers falling below 30% [24]. Additionally, elevated levels of normal protein biomarkers can occur in benign inflammatory or metabolic conditions, causing false-positive results and unnecessary medical procedures [25,26]. These limitations, along with the invasiveness and sampling variability in tissue biopsies, have increased the drive for more reliable biomarker strategies.

In this context, the scope of liquid biopsy has expanded beyond traditional circulating protein markers to include a broader range of tumor-derived molecular analytes. These include cell-free DNA (cfDNA), which contains tumor-derived circulating tumor DNA (ctDNA); circulating tumor cells (CTCs); circulating nucleosomes; microbial signatures; and various RNA molecules [27]. Among these analytes, non-coding RNAs (ncRNAs) have attracted significant interest due to their important roles in gene regulation and cancer biology [28]. NcRNAs, including miRNAs, lncRNAs, circular RNAs (circRNAs), and other small RNA types, participate in almost every level of gene regulation, from transcriptional and post-transcriptional control to chromatin remodeling, mRNA stability, and translational regulation [29].

Accumulating evidence shows that dysregulation of ncRNA-centered regulatory networks is a common feature of cancer, driving tumor initiation, progression, immune evasion, metastatic dissemination, and therapy resistance [30,31]. From a systems biology perspective, perturbations in these multilayered RNA regulatory circuits can push cells into neoplastic “attractor states,” defined as stable, self-reinforcing gene expression configurations that emerge when regulatory networks lose homeostatic control [32,33]. These attractor states reflect coordinated network-level reprogramming rather than isolated gene alterations, consistent with the view that malignant phenotypes arise from disruption of modular, interconnected regulatory systems rather than from single-gene events [32,33]. Importantly, ncRNAs show remarkable stability in biofluids because they are often protected within extracellular vesicles, bound to RNA-binding proteins, or associated with lipoprotein complexes. These features make ncRNAs especially suitable for non-invasive detection and long-term monitoring of disease [34]. Besides their diagnostic and prognostic potential, ncRNA-based therapies such as antisense oligonucleotides, miRNA mimics and inhibitors, small interfering RNAs, and aptamers are advancing through preclinical and early clinical stages, creating new opportunities for precision cancer treatment [35].

In a recent review by our group, the potential of multi-analyte liquid biopsy strategies that combine circulating miRNAs (c-miRNAs) with cell-free DNA (cfDNA) and proteins for cancer diagnosis, prognosis, and disease monitoring was examined [36]. The current narrative review builds on and broadens that work in several important ways. First, it expands the ncRNA landscape beyond miRNAs to include lncRNAs, circRNAs, and other emerging ncRNA types. Second, it incorporates these ncRNA classes within regulatory network and attractor-state frameworks, providing a system-level view of cancer biology. Third, it evaluates the use of ncRNAs as both individual biomarkers and components of multi-analyte liquid biopsy panels. This review is timely given the rapid growth of ncRNA research, advances in multi-omics technologies, and increasing interest in integrated biomarker strategies, all of which call for an updated and more comprehensive synthesis of the field. Specifically, we provide an integrated overview of ncRNA biogenesis and function, conventional cancer biomarkers and their limitations, ncRNA regulatory networks and cancer attractor states, and the application of circulating ncRNAs (c-ncRNAs) across diverse biofluids and clinical settings. Finally, we also highlight the methodological, standardization, and validation challenges that must be overcome to fully realize the potential of ncRNA-based liquid biopsy in precision oncology. The potential of ncRNAs as therapeutic agents is beyond the scope of this review and will not be discussed here.

## 2. Biogenesis and Functional Roles of Major Non-Coding RNA Classes

A clear understanding of the main ncRNA classes is crucial for interpreting their roles in cancer biology and their potential as circulating biomarkers. These classes differ significantly in their biogenesis, molecular stability, intracellular localization, and mechanisms of action. For instance, miRNAs mainly serve as post-transcriptional repressors via RNA-induced silencing complexes, lncRNAs have diverse regulatory functions at transcriptional and epigenetic levels, and circRNAs are distinguished by their covalently closed structure, which provides increased stability and allows them to act as molecular sponges or scaffolds. These differences affect not only their roles in gene regulatory networks but also their detectability and effectiveness as liquid biopsy biomarkers. In cancer, abnormal regulation of specific ncRNA classes disrupts transcriptional and post-transcriptional control, driving the development of atypical regulatory states that promote tumor initiation, progression, metastasis, and therapeutic resistance. Additionally, the inherent stability and extracellular release of many ncRNAs justify their use as minimally invasive biomarkers. Collectively, these features offer a functional basis for understanding how different ncRNA classes influence regulatory network dynamics and their application in liquid biopsy.

### 2.1. The Non-Coding Transcriptome: Housekeeping and Regulatory Non-Coding RNAs

**Figure 1:**
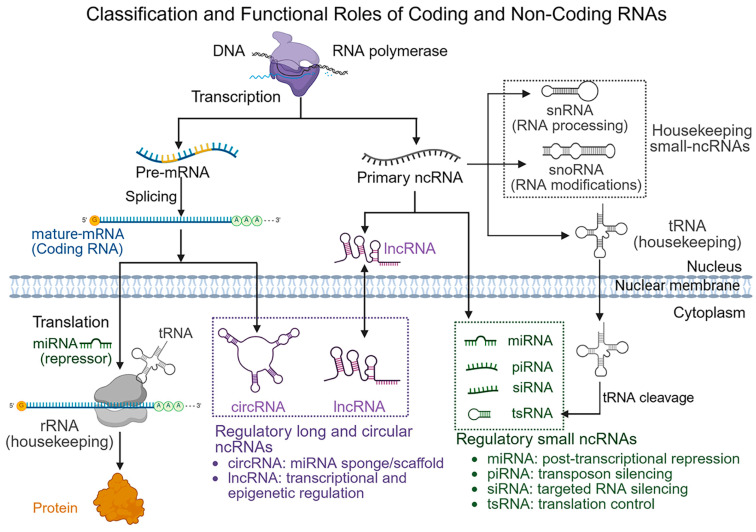
Classification and functional roles of coding and non-coding RNAs. Transcription of genomic DNA produces both protein-coding transcripts and non-coding RNAs (ncRNAs). Pre-mRNA undergoes splicing to generate mature mRNA, which is translated into protein in the cytoplasm. In parallel, transcription generates diverse ncRNA species. Housekeeping ncRNAs, including rRNA, tRNA, snRNA, and snoRNA, participate in essential cellular processes such as translation, RNA processing, and RNA modification. Regulatory ncRNAs include small RNAs (miRNAs, siRNAs, piRNAs, and tsRNAs) and long RNAs (lncRNAs and circRNAs), which regulate gene expression through mechanisms such as post-transcriptional repression, transposon silencing, RNA interference, translational control, and epigenetic regulation. This schematic illustrates canonical RNA classes, biogenesis pathways, and functions in a generalized cellular context and is not meant to represent tissue-specific expression patterns or the distribution of anatomical details. Created in BioRender. Papaneophytou, C. (2026) https://BioRender.com/3btlrke accessed on 16 March 2026.

Large-scale transcriptomic efforts, such as the Encyclopedia of DNA Elements (ENCODE) [37] and the Functional Annotation of the Mammalian Genome (FANTOM) [38], have fundamentally changed our understanding of the human transcriptome by showing that although about 75–85% of the genome is transcribed, only 1.5–2% encodes proteins. The rest of the transcriptional output includes a wide and varied array of non-coding transcripts that were once thought to be transcriptional noise but are now recognized as important regulators of gene expression and cellular homeostasis [39], as illustrated in Figure 1.

In detail, based on their biological roles, ncRNAs can broadly be divided into two major categories:

- Housekeeping ncRNAs: This class of ncRNAs is abundantly and ubiquitously expressed in cells and mainly supports essential cellular processes necessary for basic cell function and survival [40]. Among the earliest discovered RNA species, housekeeping ncRNAs have been widely studied and include ribosomal RNAs (rRNAs), transfer RNAs (tRNAs), small nuclear RNAs (snRNAs), small nucleolar RNAs (snoRNAs), and the telomerase RNA component. These molecules are generally small, typically ranging from about 50 to 500 nucleotides, and are constantly expressed across most cell types [41]. Their classical roles include rRNAs and tRNAs in protein synthesis, snRNAs in pre-mRNA splicing, and snoRNAs in RNA modification and ribosome biogenesis. Interestingly, emerging evidence suggests that some housekeeping ncRNAs can also generate regulatory molecules via specific cleavage events. For example, tRNA-derived fragments (tRFs) and tRNA halves (tiRNAs) have recently been identified as classes of small regulatory ncRNAs derived from mature or precursor tRNAs [42].
- Regulatory ncRNAs: This class of ncRNAs represents a diverse group of RNA molecules that act as key regulators of gene expression at multiple levels, including epigenetic, transcriptional, and post-transcriptional regulation [43]. Based on transcript length, regulatory ncRNAs are usually categorized into small non-coding RNAs (sncRNAs), which are typically shorter than 200 nucleotides, and long non-coding RNAs (lncRNAs), that are longer than 200 nucleotides. Main types of small regulatory ncRNAs include miRNAs, small interfering RNAs (siRNAs), and piwi-interacting RNAs (piRNAs). However, some ncRNA species can vary in length or overlap, making strict classification challenging. For example, promoter-associated transcripts (PATs) and enhancer RNAs (eRNAs) can differ in size and may belong to different ncRNA categories depending on their transcriptional context [44,45]. PATs are short, bidirectionally transcribed RNAs originating near transcription start sites, whereas eRNAs are generally low-abundance, often unstable transcripts produced from active enhancer regions [44,45]. CircRNAs are a distinct class of regulatory ncRNAs characterized by a covalently closed-loop structure formed through back-splicing. This circular shape provides exceptional resistance to exonuclease-mediated degradation, enhancing their stability in cells and biofluids [46].

These ncRNA classes differ substantially in their biogenesis, molecular functions, and patterns of dysregulation in cancer, as summarized in Table 1. It should be noted that the distinction between housekeeping and regulatory ncRNAs is not always absolute. Several RNA species traditionally classified as housekeeping molecules, such as snoRNAs and snRNAs, have recently been shown to participate in gene regulatory processes and cancer-associated transcriptomic alterations. Similarly, tsRNAs originate from housekeeping tRNAs but function as regulators of gene expression Furthermore, this classification framework has inherent limitations because ncRNA functions are highly context-dependent and can differ across cell types, developmental stages, and disease states. Length-based categorization (e.g., small versus long ncRNAs) also does not fully capture functional diversity, as molecules of similar sizes may have different regulatory roles. These points highlight the dynamic, interconnected, and context-specific nature of the non-coding transcriptome, suggesting that rigid classification schemes might oversimplify the complex functions of ncRNAs.

The following sections explore the structure and formation of different types of regulatory ncRNAs, along with a brief discussion of their role in cancer development.

### 2.2. MicroRNAs (miRNAs): Master Post-Transcriptional Regulators

MiRNAs are small, single-stranded RNA molecules, approximately 19–25 nucleotides long, that mainly regulate gene expression after transcription. They are the most widely studied class of ncRNAs in cancer biology and biomarker research [49].

Canonical miRNA biogenesis begins with transcription of miRNA genes, typically by RNA polymerase II, generating long primary transcripts (pri-miRNAs) containing characteristic hairpin structures [50,51]. In the nucleus, the microprocessor complex (Drosha–DGCR8) cleaves pri-miRNAs into ~60–70 nucleotide precursor hairpins (pre-miRNAs) [52]. These are exported to the cytoplasm via Exportin-5, where Dicer processes them into ~22-nucleotide miRNA duplexes [53]. One strand (the guide strand) is incorporated into an Argonaute (AGO) protein, most commonly AGO2, forming the RNA-induced silencing complex (RISC), while the passenger strand is typically degraded [54]. In addition to this canonical pathway, non-canonical routes exist, including Drosha-independent mirtrons and Dicer-independent processing (e.g., miR-451) [55,56].

Within RISC, miRNAs regulate gene expression by binding complementary sequences in target mRNAs, usually within the 3′ untranslated region (3′ UTR). Target recognition is primarily mediated by the seed region (nucleotides 2–8). Depending on the degree of complementarity, this interaction leads to translational repression, mRNA destabilization, or direct degradation. Because individual miRNAs can regulate multiple targets, and transcripts may be controlled by multiple miRNAs, these molecules form highly interconnected regulatory networks that fine-tune gene expression [57].

The involvement of miRNAs in cancer was first demonstrated in 2002 by Calin et al., when miR-15a and miR-16-1 were found to be deleted or downregulated in chronic lymphocytic leukemia (CLL) [58]. Since then, miRNAs have been broadly classified as oncogenic miRNAs (oncomiRs), which promote tumorigenesis when overexpressed, or tumor-suppressive miRNAs, which restrain malignant phenotypes [59]. For example, the miR-17–92 cluster is frequently amplified and promotes proliferation and apoptosis resistance by targeting tumor suppressors such as PTEN and BIM [60] whereas the let-7 family suppresses oncogenes, including RAS, MYC, and HMGA2 [61].

Large-scale miRNA profiling studies have demonstrated that miRNA signatures can accurately classify tumor types, molecular subtypes, and differentiation states, in some cases outperforming mRNA-based classifiers [62,63,64]. Moreover, the remarkable stability of circulating miRNAs (c-miRNAs) in biofluids has positioned them as promising candidates for liquid biopsy-based cancer diagnostics and disease monitoring [65]. However, despite their clinical potential, several limitations remain. These include variability in pre-analytical conditions, lack of standardized normalization strategies, and challenges in distinguishing tumor-derived miRNAs from those originating from non-malignant tissues. In addition, overlapping expression patterns across different diseases may limit their specificity when used as standalone diagnostic biomarkers [66].

### 2.3. Long Non-Coding RNAs (lncRNAs): Multifunctional Regulators of Gene Expression

LncRNAs are defined as RNA transcripts longer than 200 nucleotides that lack significant protein-coding potential [44]. Notably, the human genome is estimated to encode between 16,000 and 100,000 lncRNA genes, depending on annotation criteria, making lncRNAs the largest and most structurally diverse class of ncRNAs [67].

Most lncRNAs are transcribed by RNA polymerase II and undergo co-transcriptional processing similar to mRNAs, including 5′ capping, splicing, and 3′ polyadenylation [68]. Nevertheless, notable exceptions exist. Some lncRNAs are transcribed by RNA polymerase III, while others are produced through alternative processing pathways such as back-splicing or RNase P cleavage. Additionally, certain lncRNAs have non-canonical 3′ end structures, including small nucleolar RNA-capped termini or triple-helix stabilization motifs, which protect them from degradation [69]. LncRNAs are usually classified based on their genomic location relative to protein-coding genes as intergenic (lincRNAs), intronic, antisense, bidirectional, or enhancer-associated RNAs (eRNAs) [70]. Compared to mRNAs, lncRNAs generally show lower expression levels, more specific tissue- and cell-type patterns, and less evolutionary conservation. They are often expressed at just a few copies per cell, have highly restricted spatial and temporal expression profiles, and evolve more quickly than protein-coding genes, leading to limited sequence conservation across species [44]. However, their promoter regions and secondary structural elements are often conserved, suggesting functional importance constraint [71].

LncRNAs exert their regulatory functions through several molecular mechanisms that can be broadly grouped into four main types: signals, decoys, guides, and scaffolds. As signals, lncRNAs indicate specific transcriptional states or developmental contexts, exemplified by XIST, which mediates X-chromosome inactivation. As decoys, they sequester regulatory molecules, such as transcription factors (TFs) and microRNAs; for instance, the pseudogene-derived lncRNA PTENP1 functions as a ceRNA that sponges miRNAs targeting the tumor suppressor PTEN. As guides, lncRNAs recruit chromatin-modifying complexes to specific genomic locations, as shown by *HOX* antisense intergenic RNA (HOTAIR), which interacts with the Polycomb Repressive Complex 2 (PRC2) to silence gene expression in trans. Lastly, as scaffolds, lncRNAs serve as structural platforms for assembling multi-protein complexes; NEAT1, for example, plays a key role in the formation and organization of nuclear paraspeckles [72,73,74]. Importantly, these functional modes are not mutually exclusive, and many lncRNAs operate through multiple mechanisms depending on cellular context and subcellular localization.

Dysregulation of lncRNA expression has been reported across nearly all major cancer types, where these molecules influence tumor initiation, progression, metastasis, and therapeutic resistance [75]. For example, Metastasis-Associated Lung Adenocarcinoma Transcript 1 (MALAT1) is overexpressed in many solid tumors and promotes metastasis by regulating alternative splicing and the expression of epithelial-to-mesenchymal transition (EMT)-related genes [76]. Similarly, overexpression of HOTAIR in breast cancer reprograms the chromatin landscape by recruiting PRC2, thereby silencing metastasis-suppressor genes [77]. Nuclear Enriched Abundant Transcript 1 (NEAT1) is involved in tumor progression and chemoresistance by controlling paraspeckle-mediated sequestration of tumor-suppressive RNAs [78]. In ovarian cancer, several lncRNAs, such as HOTAIR, TP73-AS1, LINC01210, and LINC00702, are aberrantly expressed compared with normal ovarian epithelium and play roles in key malignant processes, including epithelial–mesenchymal transition, DNA damage response, angiogenesis, and uncontrolled proliferation, acting as either oncogenic drivers or tumor-suppressive regulators (reviewed in [79]). Notably, one of the few lncRNAs that has reached clinical application isProstate Cancer Antigen 3 (PCA3), which forms the basis of a U.S. Food and Drug Administration (FDA) -approved urine-based diagnostic test for prostate cancer [80]. The clear tissue specificity and regulatory versatility of many lncRNAs make them strong candidates for cancer-type-specific biomarkers and potential therapeutic targets, especially within the growing field of liquid-biopsy-based diagnostics [81].

### 2.4. Circular RNAs (circRNAs): Stable Regulators in Cancer Signaling

CircRNAs are a class of covalently closed, single-stranded RNA molecules generated through a non-canonical splicing event known as back-splicing, in which a downstream splice donor is joined to an upstream splice acceptor [82]. Typically composed of one or more exons, circRNAs are abundant in eukaryotic cells and display tightly regulated, temporal, tissue-specific, and disease-associated expression patterns. Historically, circRNAs were first identified in plant pathogens called viroids, which are small, covalently closed RNAs that infect higher plants [83].

CircRNA biogenesis usually involves complementary intronic sequences, especially Alu elements in the human genome, which bring splice sites close together through base pairing. Alternatively, RNA-binding proteins such as Quaking (QKI) and Muscleblind (MBL/MBNL1) can promote circRNA formation by linking flanking introns. Based on their structure and location, circRNAs are generally classified into three main types: exonic circRNAs (ecircRNAs), which contain only exonic sequences and mainly localize to the cytoplasm; exon–intron circRNAs (EIciRNAs), which retain intronic sequences and are typically found in the nucleus; and circular intronic RNAs (ciRNAs), which originate from intronic lariat structures. The formation of circRNAs often competes with the standard linear splicing of the same precursor mRNA, and the balance between producing circular or linear isoforms is regulated by both cis-acting sequence elements and trans-acting factors [82,84].

The functional repertoire of circRNAs has significantly expanded in recent years. One of the most well-studied functions is microRNA sponging, in which circRNAs with multiple miRNA-binding sites sequester specific miRNAs, thereby relieving repression of their target mRNAs. A well-known example is CDR1as (ciRS-7), which has over 70 conserved binding sites for miR-7 and influences miR-7 activity in neuronal and cancer-related contexts [85]. In addition to acting as miRNA sponges, circRNAs can also interact with RNA-binding proteins, serving as molecular scaffolds or decoys that control protein activity and localization [86]. Nuclear-localized EIciRNAs and ciRNAs can also regulate the transcription of their parental genes through interactions with U1 small nuclear ribonucleoprotein (U1 snRNP) and RNA polymerase II [87]. Furthermore, emerging evidence shows that some circRNAs can encode short peptides through cap-independent translation mechanisms driven by internal ribosome entry sites (IRES) or N6-methyladenosine (m6A) modifications, challenging the traditional view of circRNAs as strictly non-coding molecules [88].

CircRNA expression is often dysregulated in cancer and plays a role in various aspects of tumor biology. For example, circHIPK3 is upregulated in several cancers, including lung cancer, bladder cancer, hepatocellular carcinoma, colorectal cancer, osteosarcoma, glioma, and prostate cancer [89] and enhances cell proliferation by sponging tumor-suppressive miRNAs, including miR-124, miR-558, and miR-654 [90]. Conversely, circFOXO3 has been reported to act as a tumor suppressor by interacting with the cell cycle regulators p21 and CDK2, thereby preventing cell cycle progression [91]. Importantly, the covalently closed loop structure of circRNAs, which lacks free 5′ and 3′ ends as well as 5′ caps and 3′ poly(A) tails, offers significant resistance to exonuclease-mediated degradation. This enhanced stability, both inside cells and in external environments, makes circRNAs particularly attractive as liquid biopsy-based cancer biomarkers and potential therapeutic targets [92].

Emerging clinical studies increasingly support the translational potential of circulating circRNAs (c-circRNAs) as cancer biomarkers, due to their high stability, abundance in biofluids, and cancer-specific dysregulation [93,94]. Multiple studies have indicated that individual circRNAs can differentiate cancer patients from healthy controls with promising diagnostic accuracy. For instance, circSMARCA5 has shown diagnostic and prognostic value in gastric cancer [95]. Likewise, circ-KLDHC10 and other serum-detectable circRNAs have been reported to differentiate colorectal cancer patients from non-cancer individuals [96]. Beyond individual molecules, panels of c-circRNAs have shown promise for early detection, prognosis, and treatment monitoring across various cancer types, including breast, lung, and CRC, with multi-circRNA signatures providing better diagnostic performance than single markers in small clinical cohorts. However, these panels are still largely experimental and need more validation (reviewed in [97]).

Despite these promising findings, clinical use remains limited. Current research is restricted by small sample sizes, varied methods, and the lack of standardized protocols for sample processing, detection, and normalization. Additionally, no large prospective multicenter validation studies have confirmed the reliability of circRNA-based assays, and pre-analytical variability continues to affect reproducibility across labs. As highlighted in recent reviews [94,98], these challenges must be addressed before circRNAs can transition from promising research tools to clinically actionable biomarkers in precision oncology.

### 2.5. PIWI-Interacting RNAs (piRNAs): Guardians of Genome Stability

PiRNAs are a unique class of small non-coding RNAs, usually 24–32 nucleotides in length, that bind to PIWI-clade Argonaute proteins (PIWIL1–PIWIL4 in humans) [99]. Unlike miRNAs, piRNAs are generated through Dicer-independent pathways. Their biogenesis begins with long precursor transcripts derived from genomic piRNA clusters, which are processed by the mitochondrial endonuclease Zucchini (PLD6 in mammals) and further trimmed to yield mature piRNAs [100]. In germline cells, a secondary amplification mechanism known as the ping–pong cycle reinforces piRNA production and transposon silencing [101]. Mature piRNAs are characterized by a strong preference for uridine at the 5′ end and a 2′-O-methyl modification at the 3′ end, which enhances their stability [102].

The canonical function of the piRNA–PIWI pathway is the suppression of transposable elements in germline cells through both transcriptional silencing, often involving DNA methylation, and post-transcriptional cleavage of transposon transcripts [103]. Increasing evidence, however, suggests that piRNAs also exert regulatory roles in somatic tissues, including modulation of mRNA stability, chromatin organization, and genome integrity [104].

Aberrant expression of piRNAs and PIWI proteins has been reported in multiple cancer types, including gastric, colorectal, breast, and lung cancers. For example, piR-823 promotes gastric cancer cell proliferation through upregulation of DNA methyltransferases and has also been detected at altered levels in the circulation of cancer patients, supporting its biomarker potential [105]. Similarly, piR-651 is overexpressed in several solid tumors and promotes proliferation by regulating cell cycle-related proteins such as cyclin D1 and CDK4 [106]. Although the role of piRNAs in cancer is increasingly recognized, their functions in somatic tissues and their translational potential remain less well characterized than those of miRNAs, lncRNAs, and circRNAs [107].

### 2.6. Other Small ncRNAs: Emerging Players in Cancer Biology

Several additional classes of sncRNAs have been implicated in cancer biology and are increasingly recognized as potential biomarkers. Among these, snoRNAs, transfer tsRNAs, and snRNAs have attracted growing attention due to their regulatory roles in RNA processing and gene expression [108].

SnoRNAs are usually 60–300 nucleotides long and primarily guide the chemical modification in rRNAs, snRNAs, and other RNA substrates through 2′-O-methylation (mediated by C/D box snoRNAs) or pseudouridylation (mediated by H/ACA box snoRNAs). Besides these traditional roles in RNA modification, snoRNAs have also been shown to regulate alternative splicing, affect mRNA 3′ end processing, and act as precursors for miRNA-like small RNAs called snoRNA-derived RNAs (sdRNAs) [109]. Dysregulation of specific snoRNAs has been reported in several cancers, including SNORD78 in lung cancer, SNORA42 in prostate cancer, and SNORD114 in leukemia, suggesting potential roles in tumorigenesis and disease progression (reviewed in [110]).

TsRNAs are an emerging class of regulatory RNAs. These molecules are mainly divided into two types: tRFs, which are produced by Dicer or Angiogenin-mediated cleavage of mature tRNAs, and tRNA halves (tiRNAs), which result from cleavage of the anticodon loop of tRNAs, usually during cellular stress [111]. TsRNAs are abundant in biological fluids and are highly stable at the molecular level. Functionally, they regulate gene expression through various mechanisms, including AGO-dependent pathways similar to miRNA regulation, effects on ribosome biogenesis, and displacement of RNA-binding proteins [111,112]. Dysregulated expression of specific tRFs has been observed in breast, colorectal, and prostate cancers, and their presence in extracellular vesicles highlights their potential as circulating biomarkers [113].

SnRNAs, which include U1, U2, U4, U5, and U6, form the core of the spliceosome and are crucial for pre-mRNA splicing. Although historically considered housekeeping RNAs, snRNAs are now seen as contributors to cancer-related changes in gene expression. Recent research shows that mutations or abnormal expression of snRNAs can disrupt overall splicing patterns and encourage oncogenic transformation. For instance, recurrent U1 snRNA mutations have been found in chronic lymphocytic leukemia and hepatocellular carcinoma, causing widespread abnormal splicing events that promote tumor development [114,115].

## 3. Non-Coding RNA Regulatory Networks and Cancer Attractor States

Beyond their individual regulatory functions, ncRNAs function within highly interconnected gene regulatory networks that regulate cellular homeostasis and impact cell fate decisions [39]. In cancer, genetic mutations, epigenetic changes, and microenvironmental stresses disrupt these networks, resulting in widespread transcriptomic reprogramming that can stabilize cells in abnormal phenotypic states [116]. This section explores the architecture and topological features of ncRNA regulatory networks, explains how they are disrupted in cancer, and presents the attractor-state framework as a system-level model to understand how perturbations in ncRNA networks promote and maintain malignant phenotypes. These ideas lay the groundwork for assessing the potential of ncRNAs as network-based biomarkers and therapeutic targets in the upcoming sections.

### 3.1. Architecture of Non-Coding RNA Regulatory Networks

Gene expression in mammalian cells is regulated by complex, highly interconnected layers of control that extend beyond the traditional model of TF-mediated regulation. TFs are sequence-specific DNA-binding proteins that control the transcription of genetic information from DNA to mRNA [117]. In addition to these regulators, ncRNAs serve as key components of gene regulatory networks, coordinating gene expression at transcriptional, post-transcriptional, and epigenetic levels [118]. Understanding the topology and dynamics of these networks is crucial for explaining how localized molecular disturbances can spread through regulatory circuits and ultimately lead to the large-scale phenotypic changes characteristic of malignant transformation [119].

#### 3.1.1. MiRNA-Mediated Regulatory Networks

MiRNAs are among the most extensively studied components of ncRNA regulatory networks. A single miRNA can regulate hundreds of mRNA targets simultaneously, while individual mRNAs are often co-regulated by multiple miRNAs, forming highly interconnected bipartite regulatory networks [120]. These interactions are often organized into recurring regulatory motifs involving miRNAs and TFs. Both TFs and miRNAs serve key roles in controlling essential biological processes, such as cell proliferation, differentiation, and apoptosis. As a result, disruption of TF–miRNA regulatory networks has been linked to the development of many diseases, including cancer [118]. As essential regulators of gene expression, TFs and miRNAs frequently co-regulate target genes through regulatory motifs like feedback loops (FBLs) and feedforward loops (FFLs) [121].

In a typical TF-miRNA FBL, a TF activates the expression of a miRNA, which then represses the TF itself (or vice versa), forming a bistable regulatory circuit that can switch cells between different transcriptional states. A well-known example is the miR-200/ZEB1 double-negative feedback loop, which controls the transition between epithelial and mesenchymal cell states, a process that is central to cancer metastasis and therapeutic resistance [122].

In a TF–miRNA FFL, a TF simultaneously activates both a target gene and a miRNA that represses that same target, helping to buffer noise and control gene expression over time [123]. This regulatory motif can attenuate stochastic signaling noise and help maintain homeostatic steady-state levels of the target protein by adjusting target translation in a direction opposite to that of the input signal [124]. A well-known example is the c-Myc/miR-17-92/E2F1 circuit, where c-Myc transcriptionally activates the miR-17-92 cluster, and miRNAs derived from this cluster regulate E2F1 expression and its downstream proliferation program [125].

#### 3.1.2. Competing Endogenous RNA Networks

An additional layer of post-transcriptional regulation is provided by ceRNA networks [126]. The ceRNA hypothesis, first proposed by Salmena et al. [127], suggests that RNA transcripts sharing common miRNA response elements (MREs) can regulate one another by competing for a limited pool of miRNAs. Under this framework, diverse RNA molecules, including lncRNAs, circRNAs, pseudogene transcripts, and mRNAs, can function as miRNA sponges. Changes in the abundance of any single ceRNA may therefore redistribute miRNA availability across the network, modulating the expression of other transcripts that share the same regulatory miRNAs [128].

A well-known example is the PTEN–PTENP1 regulatory axis. The PTENP1 pseudogene transcript contains multiple MREs shared with the PTEN tumor suppressor mRNA. By sequestering regulatory miRNAs such as miR-19b, miR-20a, and miR-21, PTENP1 can relieve miRNA-mediated repression of PTEN, thus modulating tumor suppressor signaling pathways [129].

In cancer, copy-number alterations or transcriptional silencing of ceRNA components can free miRNAs to suppress other targets, thereby enhancing oncogenic signaling through indirect network mechanisms [130]. Genome-wide analyses have identified thousands of potential ceRNA interactions, and disruptions in these networks have been linked to changes in proliferation, apoptosis, and metastatic potential across various tumor types [131].

Despite the conceptual appeal of the ceRNA hypothesis, its physiological relevance in vivo remains a topic of active debate [132,133]. A major limitation stems from strict stoichiometric requirements: effective competition for shared miRNAs usually requires very high ceRNA levels relative to both the miRNA and its other targets, a condition rarely observed under normal expression levels [133,134]. Consequently, many suggested ceRNA interactions may lack sufficient molecular balance to produce noticeable regulatory effects in living organisms [134]. Furthermore, a large proportion of reported ceRNA interactions derives from computational predictions or overexpression systems, which can artificially increase apparent crosstalk and may not reflect native regulatory dynamics [132]. As a result, only a subset of ceRNA interactions, usually those involving unusually abundant transcripts or highly concentrated local microenvironments, are likely to be functionally significant in vivo [133]. These considerations highlight the importance of thorough experimental validation, quantitative modeling, and careful interpretation when assessing ceRNA-mediated regulatory networks [132,133,134].

#### 3.1.3. Long Non-Coding RNA-Mediated Chromatin Regulatory Networks

Beyond their roles as ceRNAs, lncRNAs participate in higher-order regulatory networks by recruiting and directing chromatin-modifying complexes to specific genomic loci [40]. For example, HOTAIR interacts simultaneously with both the Polycomb Repressive Complex 2 (PRC2) and the LSD1/CoREST/REST complex, coordinating multiple chromatin-modifying activities and helping to silence metastasis-suppressor genes in trans [135].

Similarly, XIST orchestrates the silencing of an entire chromosome by sequentially recruiting multiple epigenetic regulatory complexes in a spatially organized manner [136]. Through such interactions, lncRNAs establish epigenetic regulatory networks that operate on broader genomic scales and longer timescales than post-transcriptional miRNA-mediated networks. In cancer, aberrant expression of chromatin-associated lncRNAs can reprogram epigenetic landscapes, promoting dedifferentiated or stem-like transcriptional states that facilitate tumor initiation and progression [137,138].

#### 3.1.4. Circular RNA Network Integration

CircRNAs further complicate ncRNA regulatory networks through several complementary mechanisms. Many circRNAs act as miRNA sponges, directly participating in ceRNA regulatory circuits. Their exceptional stability enables them to maintain ongoing regulatory effects on miRNA availability and subsequent gene expression [139].The most extensively studied example is CDR1as (ciRS-7), which contains more than 70 binding sites for miR-7. By sequestering miR-7, CDR1as modulates the expression of multiple miR-7 target genes involved in neuronal signaling and cancer-related pathways, including the EGFR/PI3K/AKT, RAF/MEK/ERK, and NF-κB signaling cascade [140].

In addition, circRNAs can interact with RNA-binding proteins or regulate transcription of their parental genes through mechanisms involving exon–intron circRNAs (EIciRNAs), thereby establishing local feedback loops between circular and linear RNA isoforms [141]. Although the full integration of circRNAs into ncRNA regulatory networks remains an area of active investigation, accumulating evidence indicates that circRNA–miRNA–mRNA regulatory axes represent important functional modules in multiple cancer types, including breast, colorectal, lung, bladder, glioma, melanoma, and esophageal cancers [142].

### 3.2. Network Topology and Properties

The structure of ncRNA regulatory networks displays several features typical of complex biological systems. Large-scale network reconstruction studies that integrate miRNA–target databases, lncRNA interaction catalogs, and experimentally validated ceRNA interactions have revealed recurring topological traits that shape how regulatory signals propagate through these networks [143].

One key feature of ncRNA regulatory networks is their scale-free architecture, where connections distribute approximately according to a power law. In these networks, most nodes (genes, miRNAs, or lncRNAs) have relatively few interactions, while a small number of highly connected hub nodes represent many regulatory links. This design offers robustness against random disturbances, but makes the network susceptible to targeted attacks on these hubs [144].

Several hub miRNAs, including miR-21, miR-155, and members of the let-7 family, occupy highly connected positions within miRNA regulatory networks, regulating hundreds of target transcripts across multiple signaling pathways. These miRNAs are often dysregulated in various cancer types, including breast, lung, colorectal, gastric, hepatocellular, pancreatic, and prostate cancers, linking network centrality to widespread transcriptional reprogramming and disease progression [145,146]. Due to this topology, perturbing hub or bottleneck nodes through mutation, epigenetic change, or copy number variation can cause cascading effects throughout the regulatory network by disrupting information flow. In contrast, perturbations that affect peripheral nodes are often buffered by redundant interactions and the network’s plasticity [147,148].

Another key feature of ncRNA regulatory networks is modularity, where regulatory interactions are organized into functionally cohesive clusters of densely connected nodes that relate to specific biological processes or pathways. These modules represent coordinated regulatory programs that control processes such as cell cycle progression, EMT, immune signaling, or metabolic reprogramming [119,149].

In cancer research, module-level analyses often provide more insight than single-gene approaches for identifying driver alterations and predicting patient outcomes [150]. For example, Pepe et al. [151] examined miRNA–mRNA co-expression relationships across 13 cancer types using thousands of experimentally validated interactions and demonstrated a widespread loss of coordinated miRNA–mRNA regulation in tumors. The disrupted interaction sets were enriched for pathways associated with (EMT, immune evasion, and metabolic adaptation, consistent with extensive evidence that miRNAs regulate these processes through coordinated network programs.

NcRNA regulatory networks are highly interconnected and dominated by regulatory hubs, allowing signals to spread efficiently throughout the network. Coupled with scale-free hub connectivity, this structure helps explain the widespread downstream effects seen when individual ncRNAs are experimentally altered. Altering the expression of a single miRNA or lncRNA can simultaneously influence multiple distant pathways, contributing to the pleiotropic phenotypes often observed in cancer cells [32].

### 3.3. The Cancer Attractor-State Framework

The topological features described above provide a structural basis for how perturbations in ncRNA regulatory networks can facilitate large-scale phenotypic transitions during tumorigenesis. The topological features described above provide a structural basis for how perturbations in ncRNA regulatory networks can facilitate large-scale phenotypic transitions during tumorigenesis. The conceptual framework used to interpret these transitions derives from dynamical systems theory and the application of attractor-state models to gene regulatory networks [152,153]. In this view, a cell’s transcriptome represents a point in a high-dimensional state space (with each axis representing an individual gene), and the dynamics of regulatory interactions restrict cells to a limited set of stable configurations (attractors) that correspond to distinct cellular phenotypes or functional states [153].

This picture parallels Waddington’s epigenetic landscape: attractors are basins in the landscape, and developmental or pathological trajectories reflect movement toward particular basins [154,155]. Genetic and epigenetic alterations can deform the regulatory landscape, increasing the probability that cells occupy aberrant, self-sustaining attractors that support malignant traits rather than simply producing linear, reductionist changes in single genes [153,155,156].

Neoplastic attractors are defined by self-sustaining gene expression programs that underpin hallmark cancer behaviors, including persistent growth, resistance to cell death, metabolic reprogramming, and invasive potential. The attractor framework helps clarify several otherwise confusing observations in oncology: phenotypic convergence (different tumors with similar transcriptomes), phenotypic plasticity (reversible EMT and hybrid states), and the relatively small number of stable molecular subtypes within many cancers despite high mutational diversity [157].

Mechanistic roles of ncRNAs in attractor dynamics ncRNAs occupy central, high-influence positions within gene regulatory networks and therefore can bias attractor-state distributions through multiple mechanisms:

- MiRNA–transcription factor bistable loops: Circuits such as the miR-200/ZEB1 and miR-34/SNAIL modules function as bistable or multistable switches that define epithelial, mesenchymal, and hybrid states; dysregulation of these loops lowers the effective barrier between states and promotes transitions associated with metastasis and therapy resistance [122].
- CeRNA interactions establish long-range dependencies in post-transcriptional regulation. Losing or disturbing a single, influential ceRNA can change miRNA availability and, under favorable stoichiometric and affinity conditions, spread expression shifts that alter the regulatory landscape toward different attractor states. Experimental examples (e.g., PTEN/PTENP1; ciRS-7/miR-7) show that disrupting one ceRNA node can significantly affect downstream target expression, while quantitative modeling and stoichiometry studies indicate these effects depend on context. This requires specific relative abundances, binding affinities, and network topology to produce system-level outcomes [127,158,159,160].
- LncRNAs that recruit chromatin-modifying complexes can reconfigure regulatory topology by altering local chromatin states and transcriptional outputs, thereby changing which attractor basins are accessible or stable [137]. Canonical examples include HOTAIR, which recruits PRC2 and LSD1 to reprogram chromatin domains and promote metastatic gene-expression programs [77], and XIST, which orchestrates chromosome-wide silencing through PRC2-mediated deposition of repressive histone marks [161]. Similar mechanisms operate in cancer-associated lncRNAs such as ANRIL, whose recruitment of PRC1/PRC2 remodels the INK4/ARF locus, illustrating how lncRNA-driven epigenetic remodeling can stabilize aberrant transcriptional states consistent with neoplastic attractors [137,155].
- The modular organization of ncRNA regulatory networks means that collapse of intra-module connectivity or the emergence of aberrant inter-module links can reorganize network topology in cancer. Empirical reconstructions of tumor-specific ceRNA networks, such as the cervical squamous cell carcinoma (CESC) network reported by Song et al. [162] reveal the loss of normal module structure, the emergence of new subnetworks, and the rewiring of hub lncRNAs that bridge previously separate modules. Dynamical systems models predict that such topological perturbations can destabilize normal attractors and create novel, pathology-associated attractor states with no normal-tissue counterpart, providing a mechanistic explanation for the emergence of stable malignant transcriptional programs [153,156].

Recent advances in single-cell transcriptomics have provided experimental support for the attractor-state framework. Single-cell RNA sequencing (scRNA-seq) studies frequently reveal discrete transcriptional states within tumor cell populations and allow for reconstruction of transition trajectories between them [163]. Computational trajectory inference methods, including Monocle, RNA velocity, and diffusion pseudotime, have been used to map the paths by which cells traverse the gene expression landscape, identifying intermediate states that may represent saddle points between attractor basins [164]. Integration of miRNA expression profiles with state-resolved transcriptomic data indicates that miRNA regulatory activity varies across cellular states and contributes to the stabilization of state-specific gene expression programs. This is supported by cell-state–resolved miRNA profiling in the mouse immune system [165], multi-omics analyses in rat spinal cord injury models showing dynamic miRNA–mRNA regulatory modules [166], and human cancer studies demonstrating that inferred miRNA activity defines distinct tumor-specific regulatory states [167].

Complementary computational modeling approaches, including Boolean network models, ordinary differential equation systems, and stochastic simulations, have demonstrated that perturbation of ncRNA nodes within reconstructed regulatory networks can alter the distribution of attractor states in ways consistent with experimentally observed phenotypic transitions [168]. For example, Boolean network models of the miR-200/ZEB/SNAIL/miR-34 regulatory circuit predicted the existence of a hybrid epithelial/mesenchymal cellular state. This prediction was later confirmed experimentally and has been associated with increased metastatic potential and stem-like properties in cancer cells [169,170].

Because ncRNAs occupy central roles within multilayered regulatory networks that coordinate chromatin organization, transcription, RNA processing, and signaling, changes in their expression often reflect system-wide shifts in cellular identity. Their integration into these regulatory hubs, along with their stability in extracellular fluids, makes c-RNAs especially informative biomarkers capable of capturing global transcriptional reprogramming associated with tumor development and progression [171,172].

### 3.4. Clinical and Translational Implications of Network-Level Analysis

The network and attractor-state perspectives discussed above have significant implications for the development of ncRNA-based cancer diagnostics and therapies. Instead of seeing individual ncRNAs as isolated biomarkers, a system-level approach highlights the importance of network-based biomarker panels, which consist of coordinated sets of ncRNAs from the same regulatory modules or pathways (discussed further below). These multi-component signatures may offer more reliable diagnostic and prognostic information by reflecting the overall regulatory state of tumor cells rather than focusing on single molecular events [173].

From a therapeutic perspective, network analysis identifies potential intervention points within ncRNA regulatory circuits. In particular, hub nodes, feedback loops, and bistable regulatory switches represent attractive targets for therapeutic modulation. Perturbation of a key regulatory node, such as restoring a tumor-suppressive miRNA or disrupting an oncogenic ceRNA interaction, could destabilize malignant attractor states and promote transitions toward less aggressive or more therapy-sensitive phenotypic configurations [174].

Recent advances in multi-omics technologies are further improving our ability to map these regulatory networks with high resolution. Combining such datasets with computational attractor-state modeling provides a powerful approach for identifying essential regulatory nodes and predicting how targeted interventions may transform cellular state landscapes [175]. These approaches are expected to play an increasingly important role in the development of precision oncology strategies

## 4. Non-Coding RNAs as Liquid Biopsy Biomarkers

The clinical translation of ncRNA biology into actionable diagnostic tools depends on the reliable detection and quantification of tumor-associated ncRNAs in minimally invasive biospecimens [176]. As discussed above, liquid biopsy, broadly defined as the molecular analysis of tumor-derived analytes in body fluids, offers an especially attractive platform for this purpose [177]. In contrast to genomic biomarkers such as cfDNA and cellular analytes such as circulating tumor cells, ncRNAs provide unique insights into the dynamic regulatory state of tumors, reflecting ongoing transcriptional, post-transcriptional, and epigenetic processes. Their expression patterns capture functional network activity, including pathway activation, immune modulation, metabolic rewiring, EMT programs, and drug-resistance mechanisms that cannot be inferred from static genomic alterations alone. Because ncRNAs respond rapidly to microenvironmental cues and therapeutic pressure, they offer a real-time readout of tumor plasticity and adaptive behavior, complementing the mutational information obtained from cfDNA and the cellular phenotypes represented by CTCs. In addition, their stability in biofluids and enrichment in extracellular vesicles further enhance their utility as minimally invasive biomarkers for monitoring dynamic disease states and treatment response [28,173,178]. This section discusses the biological basis for the stability of ncRNAs in biofluids and reviews evidence supporting c-miRNAs, lncRNAs, circRNAs, and other small ncRNA species as biomarkers in cancer.

### 4.1. Mechanisms Underlying ncRNA Release and Extracellular Stability

Circulating tumor nucleic acids, including ncRNAs, are released from tumor cells, stromal cells, and immune cells within the tumor microenvironment through both active secretion and passive release (reviewed in [179]). Understanding these release mechanisms and the factors that confer extracellular stability is essential for interpreting ci c-ncRNA profiles in liquid biopsy applications.

Notably, circRNAs possess an intrinsic structural advantage as circulating biomarkers because their covalently closed loop structure renders them highly resistant to exonuclease-mediated degradation [24,42]. In plasma, circRNAs exhibit substantially longer half-lives than their linear counterparts, and their incorporation into extracellular vesicles may further enhance their persistence [87]. Together, these features make circRNAs among the most stable ncRNA species detectable in liquid biopsy.

**Figure 2:**
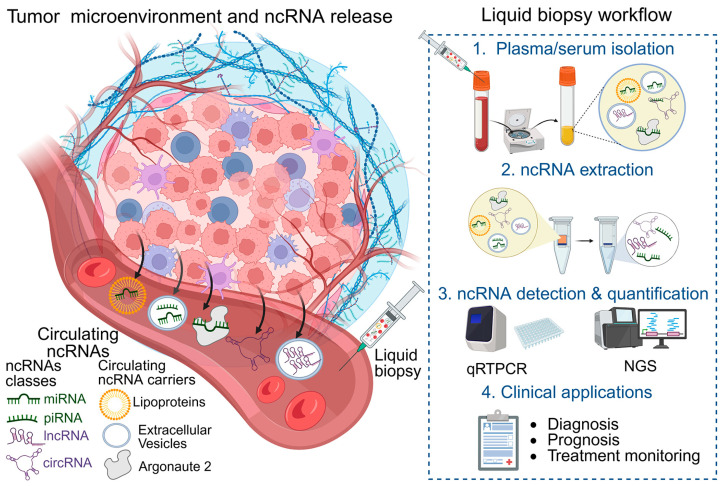
Circulating ncRNAs as liquid biopsy biomarkers in cancer. Tumor cells and components of the tumor microenvironment release ncRNAs into circulation through multiple mechanisms, including extracellular vesicles, lipoprotein complexes, and Argonaute-associated ribonucleoprotein particles. These circulating ncRNAs (c-ncRNAs) can be isolated from plasma or serum and analyzed using molecular detection platforms such as quantitative PCR or next-generation sequencing. C-ncRNAs therefore represent minimally invasive biomarkers with potential applications in cancer detection, prognosis, and treatment monitoring. The schematic illustrates the basic principles of ncRNA release and liquid biopsy analysis and does not include quantitative comparisons between clinical groups or specific measurement methodologies. Created in BioRender. Papaneophytou, C. (2026) https://BioRender.com/jdya7hd accessed on 16 March 2026.

The major routes through which tumor-associated ncRNAs enter circulation, and their subsequent analysis in liquid biopsy workflows are summarized in Figure 2 and discussed in the subsequent sections below.

#### 4.1.1. Extracellular Vesicle-Mediated Release of ncRNAs

A substantial fraction of c-ncRNAs is actively secreted within extracellular vesicles (EVs), including exosomes (30–150 nm), microvesicles (100–1000 nm), and apoptotic bodies (1000–5000 nm) [180,181]. Exosomes originate through the endosomal pathway and are released upon fusion of multivesicular bodies with the plasma membrane, while microvesicles are generated by outward budding of the cell surface [182]. The lipid bilayer of EVs shields their RNA cargo from RNase-mediated degradation and confers resistance to pH fluctuations, freeze–thaw cycles, and prolonged storage [183].

Importantly, ncRNA loading into EVs is selective rather than random; RNA-binding proteins, including hnRNPA2B1 and SYNCRIP, recognize specific sequence motifs (such as the GGAG motif in miRNAs) that guide preferential packaging, and ceramide-dependent pathways regulated by neutral sphingomyelinase 2 (nSMase2) further affect exosomal RNA content [184]. This selective sorting means that the exosomal ncRNA profile can vary greatly from the intracellular profile of the original cell, which has important implications for biomarker interpretation [180].

EV-mediated transfer of ncRNAs has been implicated in multiple aspects of tumor biology, including angiogenesis, immune modulation, pre-metastatic niche formation, and the development of therapy resistance, underscoring the functional significance of this release pathway [185]. 

#### 4.1.2. Protein-Associated Release and Transport of Circulating ncRNAs

A significant proportion of c-miRNAs exists outside vesicles, bound to AGO2 protein complexes. The AGO2-miRNA interaction, which is central to intracellular RISC function, persists in the extracellular milieu and confers remarkable resistance to RNase digestion, boiling, and extreme pH conditions [186]. Additional RNA-binding proteins, including nucleophosmin 1 (NPM1), have also been identified as extracellular carriers of specific miRNA subsets [187].

High-density lipoprotein (HDL) and low-density lipoprotein (LDL) particles carry another fraction of c-miRNAs, with HDL-associated miRNAs shown to be functionally delivered to recipient cells, where they can influence gene expression [188,189]. Notably, the lipoprotein-miRNA profile changes in cancer patients, and HDL-bound miR-223 and miR-92a have been suggested as cancer-related biomarkers. The relative contribution of vesicular, protein-bound, and lipoprotein-associated fractions varies depending on the ncRNA species and disease context, and this distribution may itself provide independent diagnostic information [28]. 

#### 4.1.3. Passive Release of ncRNAs from Dying Cells

In addition to regulated secretion through extracellular vesicles and ribonucleoprotein complexes, intracellular ncRNAs can be passively released during cellular stress and various forms of cell death. Necrosis, apoptosis, pyroptosis, ferroptosis, and NETosis can disrupt membrane integrity and alter RNA-binding protein homeostasis, thereby enabling cytosolic and nuclear ncRNAs to enter the extracellular space. Because these passive release pathways produce RNA cargoes and carriers different from those created by selective extracellular vesicle-mediated export, the resulting extracellular RNA landscape may provide mechanistic and diagnostic information about the underlying cellular processes [190].

In cancer, elevated rates of tumor cell turnover, tissue remodeling, and therapy-induced cell death are likely to contribute substantially to this pool of c-ncRNAs. Although passively released ncRNAs may lack the selective enrichment that characterizes extracellular vesicle-mediated secretion, they nevertheless contribute significantly to the overall c-ncRNA burden and may reflect tumor burden, proliferative activity, tissue damage, and treatment response [179].

### 4.2. Circulating miRNAs as Cancer Biomarkers

Following the landmark demonstration by Mitchell et al. in 2008 [35] that tumor-derived miRNAs are present in plasma in a stable and reproducible form, c-miRNAs have become the most extensively investigated class of ncRNAs in liquid biopsy research. Numerous studies have since shown that miRNAs can serve as diagnostic, prognostic, and predictive biomarkers across a wide range of cancers, with more than 4500 papers published in PubMed in 2025.

In this section, we highlight representative examples from four major cancer types: lung, colorectal, breast, and prostate cancer, which are among the most common malignancies worldwide and pose persistent challenges in early detection and disease stratification [24]. These cancers have also been the focus of extensive research on c-miRNAs, including large-scale diagnostic and prognostic studies that support their clinical relevance [191].

While the diagnostic and prognostic potential of c-miRNAs has also been widely explored in other malignancies, including ovarian cancer, hepatocellular carcinoma, gastric cancer, pancreatic cancer, and liver cancers, a detailed discussion of these diseases is beyond the scope of the present review. The selected examples, therefore, aim to illustrate the broader clinical potential of c-miRNAs rather than provide an exhaustive catalog of all reported biomarkers. A cross-cancer summary of representative c-miRNA biomarkers is provided in Table 2.

Notably, while certain c-miRNAs demonstrate strong biomarker potential as standalone markers, multi-miRNA panels and combinatorial approaches incorporating additional circulating biomarkers, including proteins and cfDNA, generally achieve higher diagnostic accuracy, improved reproducibility, and greater clinical utility. These advantages, highlighted in our recent review [36] and further discussed in Section 5 underscore the value of integrative liquid biopsy strategies.

#### 4.2.1. Lung Cancer

Lung cancer remains the leading cause of cancer-related mortality worldwide [24]. The disease is broadly classified into two major histological types: non-small-cell lung cancer (NSCLC) and small-cell lung cancer (SCLC), with NSCLC accounting for approximately 85% of all cases [200]. The current standard for lung cancer screening is low-dose computed tomography (LDCT); however, this approach has several limitations, including relatively low specificity, exposure to ionizing radiation, and substantial resource requirements for equipment and clinical personnel [201].

C-miRNA signatures have therefore emerged as promising minimally invasive biomarkers for lung cancer detection and risk stratification (reviewed in [202]). Among the most frequently reported miRNAs in NSCLC are miR-21 and miR-145, both of which have been implicated in tumorigenesis and disease progression [203,204]. Notably, miR-21 has been consistently associated with early-stage lung cancer and is considered one of the most robust c-miRNA biomarkers identified to date [204].

Several c-miRNA panels have also been proposed for the early detection of NSCLC. For example, a panel comprising miR-21, miR-126, miR-210, and miR-486-5p distinguished early-stage NSCLC from healthy controls with reported sensitivities of 73–87% and specificities of 83–97% in independent validation cohorts [192]. Similarly, the miR-Test, a 13-miRNA serum signature, was prospectively validated in the Multicenter Italian Lung Detection (MILD) trial and demonstrated the ability to identify lung cancer up to two years before CT-based diagnosis, representing one of the most advanced examples of miRNA-based early detection [193].

Beyond early detection, c-miRNAs may also assist in histological and molecular stratification of lung cancer. Several studies have demonstrated that miRNA signatures differ across lung cancer subtypes, enabling discrimination between lung adenocarcinoma (LUAD), lung squamous cell carcinoma (LUSC), and SCLC [205]. For example, miR-375, miR-203, and miR-205 have been reported to distinguish LUSC from other NSCLC subtypes [206], while machine learning approaches have identified miR-944 and miR-205 as informative markers for classifying LUAD and LUSC tumors [207]. In addition c-miRNA models incorporating miR-375-3p, miR-320b, and miR-144-3p have shown potential for identifying metastatic SCLC [208], and miR-17, miR-190b, and miR-375 have been reported to differentiate SCLC from NSCLC with an area under the curve (AUC) of 0.869 [209].

More recently, Abdipourbozorgbaghi et al. [210] demonstrated that c-miRNAs may serve as powerful biomarkers across the entire NSCLC clinical pathway. Their study identified subtype-specific diagnostic miRNA panels with high accuracy and validated these findings in an independent cohort of more than 4000 patients. In addition, several c-miRNAs functioned as independent prognostic markers, while distinct miRNA clusters predicted progression-free survival in patients receiving anti–PD-1 therapy. Longitudinal changes in c-miRNA levels also mirrored radiological treatment responses, highlighting their potential utility for real-time disease monitoring.

#### 4.2.2. Breast Cancer

Breast cancer remains one of the most common cancers and a leading cause of cancer-related death among women worldwide [24]. Mammography is currently the gold-standard screening method for breast cancer detection; however, it has several limitations, especially in younger women with dense breast tissue, where the disease can be more aggressive and harder to detect early. Traditional diagnostic methods, including imaging tests and histopathological analysis, also face challenges in early detection and accurate molecular classification [211]. Clinically, breast cancer diagnosis and classification often depend on biomarkers like changes in the breast cancer susceptibility genes (BRCA1/2), human epidermal growth factor receptor 2 (HER2), and hormone receptor status. Additionally, circulating protein biomarkers such as cancer antigen 15-3 (CA15-3) and CEA are commonly used in clinical practice, especially for disease monitoring [212,213]. However, these markers lack enough sensitivity and specificity for early diagnosis, as their levels may also be elevated in other cancers and some benign conditions [212]. Since early detection is closely linked to better clinical outcomes and long-term survival, there is a clear need for additional minimally invasive molecular biomarkers that can supplement traditional diagnostic methods.

An increasing number of studies have demonstrated that dysregulation of specific miRNAs is closely associated with breast cancer initiation, progression, invasion, and metastasis (reviewed in [214]). For example, miR-21 is frequently overexpressed in breast cancer tissues and promotes tumor cell proliferation and invasion, supporting its role as an oncogenic miRNA [215].

C-miRNAs have therefore attracted considerable attention as potential non-invasive biomarkers for breast cancer detection. A recent systematic review and meta-analysis by Garrido-Palacios et al. [194] identified 34 c-miRNAs that were consistently dysregulated across 38 high-quality studies. Among these, miR-21 and miR-155 were the most frequently reported markers with strong diagnostic potential. Their quantitative synthesis showed that miR-155 alone achieved a pooled sensitivity and specificity of 86% and 93%, respectively, with an AUC of 0.96. Furthermore, multi-miRNA panels, particularly those including miR-1246, miR-206, miR-24, and miR-373, reached sensitivities of up to 98% and specificities of up to 96%, outperforming traditional tumor markers such as CEA and CA15-3.

In an earlier study, Shimomura et al. [216] validated a five-miRNA serum panel (miR-1246, miR-1307-3p, miR-4634, miR-6861-5p, and miR-6875-5p) in a large cohort (over 4000 participants), achieving a sensitivity of 97.3%, specificity of 82.9%, and overall accuracy of 89.7% for early-stage breast cancer detection. Consistent with these findings, earlier comprehensive reviews have highlighted miR-21, miR-155, miR-10b, and miR-145 as some of the most frequently dysregulated c-miRNAs in breast cancer patients. These studies further emphasize that multi-miRNA signatures generally outperform single-miRNA assays, with many panels achieving diagnostic accuracies with an AUC greater than 0.85 when distinguishing breast cancer patients from healthy controls [195].

Importantly, specific c-miRNA profiles have also been associated with breast cancer molecular subtypes, including luminal A/B, HER2-positive, and triple-negative disease, suggesting potential for non-invasive molecular classification [217]. Additionally, c-miR-210 and c-miR-373 show therapy-associated increases and are correlated with tumor burden and stage. miR-210 has also been reported to be linked to trastuzumab resistance in an independent cohort. However, neither marker has consistently predicted pathological complete response, highlighting the need for larger prospective validation [218,219].

Other c-miRNAs have been implicated in therapy response and disease progression. For instance, miR-125b has been associated with breast cancer invasion and chemoresistance. For example, Wang et al. [220] reported that elevated circulating miR-125b levels correlate with poor chemotherapy response and increased metastatic potential, highlighting its potential as a predictive biomarker for treatment outcomes

#### 4.2.3. Colorectal Cancer

CRC is one of the most prevalent malignancies worldwide and a leading cause of cancer-related mortality, particularly in developed countries [221]. The etiology of CRC is complex and multifactorial, involving both genetic and environmental factors. Current CRC screening strategies include colonoscopy, stool-based tests, and imaging modalities such as computed tomography (CT) colonography [222]. Although colonoscopy remains the gold standard for CRC detection due to its high sensitivity and ability to remove precancerous polyps, it is invasive, costly, and often associated with patient discomfort [223]. Consequently, considerable effort has been directed toward identifying minimally invasive biomarkers for CRC detection. In this context, c-miRNAs have emerged as promising candidates for early diagnosis (reviewed in [224]).

One of the earliest c-miRNAs proposed as a CRC biomarker was miR-92a, first identified by Ng et al. [196]. Plasma miR-92a demonstrated significant diagnostic accuracy, with an area under the curve (AUC) of approximately 0.89 for distinguishing CRC patients from healthy individuals. Subsequent studies have expanded this concept by developing multi-miRNA diagnostic panels. For example, Vychytilova-Faltejskova et al. [197] identified a four-miRNA serum signature comprising miR-23a-3p, miR-27a-3p, miR-142-5p, and miR-376c-3p, which achieved an AUC of 0.917 with 89% sensitivity and 81% specificity, and maintained strong performance in early-stage disease (AUC 0.877).

C-miRNAs also show prognostic significance in CRC. Members of the miR-200 family, which take part in the miR-200/ZEB1 regulatory loop discussed in Section 3, are crucial for maintaining epithelial identity and controlling epithelial–mesenchymal transition. Among them, miR-200c has become a clinically important biomarker: serum miR-200c levels are notably higher in metastatic CRC and independently predict lymph node involvement, distant metastasis, recurrence, and overall survival. Likewise, increased circulating miR-141 levels have been linked to metastatic disease and poor prognosis in CRC patients [225,226].

Evidence from systematic reviews further supports the diagnostic potential of c c-miRNAs in CRC. A recent meta-analysis by Schwab and Nonaka [65], which included 37 studies (44 substudies) encompassing 2775 CRC patients, evaluated both blood-derived and saliva-derived miRNAs as diagnostic biomarkers. The pooled diagnostic performance was strong, with AUC values of 0.87 for combined blood- and saliva-based assays and 0.86 for blood-only assays, accompanied by pooled sensitivities of 0.76 and specificities of 0.83.

Nevertheless, some studies have highlighted potential limitations of c-miRNAs as ultra-early detection biomarkers. In a nested case–control study within the EPIC-Italy cohort, Padroni et al. [227] evaluated eight candidate miRNAs—including miR-21, miR-155, miR-92a, and miR-145—and found no significant associations with colon cancer risk when measured nearly a decade before diagnosis. These findings suggest that while circulating miRNAs show strong diagnostic potential in established disease, their ability to detect CRC during the earliest preclinical phases may be limited.

#### 4.2.4. Prostate Cancer

Prostate cancer is the second most frequently diagnosed cancer among men worldwide, after lung cancer, and represents the fifth leading cause of cancer-related mortality [228]. The disease exhibits remarkable heterogeneity in clinical progression and prognosis, both across populations and within individual patients [229]. Currently, the main screening tools for prostate cancer are digital rectal examination (DRE) and the measurement of PSA in blood [230]. However, PSA testing has important limitations, particularly its relatively low specificity, as elevated PSA levels may also occur in benign conditions such as benign prostatic hyperplasia or prostatitis, leading to false-positive results and potential overdiagnosis [231].

Increasing evidence suggests that miRNAs play important regulatory roles in prostate cancer biology, including the control of tumor growth, androgen receptor signaling, and apoptotic pathways (reviewed in [232]). Dysregulation of miRNA expression has been widely associated with prostate cancer initiation, progression, and metastasis [232,233,234].

The clinical need for better biomarkers beyond PSA has therefore led to extensive research into c-miRNAs as minimally invasive indicators for prostate cancer. Among these, miR-141 and miR-375 are consistently elevated in metastatic castration-resistant prostate cancer and have been shown to correlate with disease burden and overall survival [199]. Additionally, exosomal miR-1290 and miR-375 have been identified as independent predictors of overall survival in patients with advanced prostate cancer. When combined with clinical prognostic factors such as PSA levels and time to androgen deprivation therapy (ADT) failure, including these miRNAs improved the predictive accuracy of multivariate models, raising the AUC from 0.66 to 0.73 [198].

For diagnostic purposes, both individual c-miRNA and multi-miRNA panels have shown promising results in detecting prostate cancer. Mharrach et al. [235] reported that miR-21 and miR-221 were significantly upregulated in prostate tumor tissues compared with controls and exhibited strong diagnostic accuracy, with AUC values of 0.90 and 0.89, respectively. Notably, elevated expression of both miRNAs was associated with higher Gleason scores and more advanced tumor stage, further supporting their utility for early detection and risk stratification. In addition, a plasma four-miRNA signature consisting of miR-152-3p, miR-98-5p, miR-326, and miR-4289 demonstrated robust diagnostic performance, achieving an AUC of 0.88 for distinguishing prostate cancer patients from healthy controls [236].

Recent studies have further expanded the understanding of miRNA biomarkers in prostate cancer. Xiang et al. [237] profiled miRNA expression across localized, locally invasive, and metastatic prostate cancer tissues and identified 228 differentially expressed miRNAs, highlighting miR-6715b-3p as a consistently upregulated candidate. Functional enrichment analysis linked these miRNAs to key oncogenic pathways, including MAPK signaling, autophagy, and metabolic regulation, supporting their relevance in disease progression.

Similarly, the MCC-Spain case–control study [238] analyzed 46 circulating serum miRNAs in 203 prostate cancer patients and 54 controls, identifying 14 miRNAs significantly associated with disease status. Among these, miR-199a-5p and miR-150-5p showed consistent differential expression across all Gleason categories, whereas miR-24-3p was uniquely elevated in high-risk tumors. Predictive models based on LASSO-selected miRNA panels demonstrated strong discriminatory performance, particularly for low-risk disease (AUC = 0.930), highlighting the potential of c-miRNAs for non-invasive diagnosis and risk stratification.

### 4.3. Circulating Long Non-Coding RNAs as Cancer Biomarkers

Although c-lncRNAs have been studied less extensively than miRNAs, their tissue-specific expression patterns and detectable presence in biofluids make them attractive candidates for cancer-type-specific biomarker applications [239]. Representative c-lncRNAs examined as cancer biomarkers are summarized in Table 3 and reviewed in the following sections.

#### 4.3.1. Prostate Cancer Antigen 3 (PCA3)

PCA3 represents the most clinically advanced lncRNA biomarker identified to date [250]. PCA3 is a portion of noncoding mRNA located on chromosome 9q21–22, and it is overexpressed in over 95% of prostate cancers [251]. PCA3 is detectable in urine following digital rectal examination and received FDA approval in 2012 as an adjunct test to PSA for guiding repeat biopsy decisions [240]. The PCA3 score, defined as the ratio of PCA3 mRNA to PSA mRNA [252] demonstrates higher specificity than serum PSA alone, reducing unnecessary biopsies by approximately 50% while maintaining sensitivity for high-grade disease [253]. The clinical implementation of PCA3 illustrates that lncRNA-based biomarkers can achieve regulatory approval and clinical utility, providing a translational model for other lncRNA candidates.

#### 4.3.2. Metastasis-Associated Lung Adenocarcinoma Transcript 1 (MALAT1)

MALAT1 is one of the most well-studied lncRNAs and has played a key role in challenging the outdated idea that large non-protein-coding regions of the human genome are simply “junk DNA” [241].

Located on human chromosome 11q13, MALAT1 is detectable in plasma and serum and was initially identified as a prognostic marker for early-stage lung cancer [254]. Notably, in NSCLC, circulating MALAT1 levels are elevated compared with controls, and higher levels are associated with more advanced TNM stages, supporting its potential as a minimally invasive biomarker [242]. In addition to lung cancer, its expression has been found to be dysregulated in a wide range of malignancies, including breast, prostate, gastric, colorectal, and liver cancers, indicating a broad role in tumorigenesis and metastatic progression [241,255]. This widespread dysregulation indicates that MALAT1 could have both diagnostic and prognostic roles in cancer.

However, because MALAT1 is dysregulated across multiple tumor types, its specificity as a stand-alone diagnostic biomarker may be limited. Consequently, its greatest clinical value may lie in its incorporation into multi-analyte biomarker panels rather than use as an isolated marker.

#### 4.3.3. *HOX* Transcript Antisense RNA (HOTAIR)

HOTAIR represents a well-characterized example of an oncogenic trans-acting lncRNA [256]. HOTAIR expression is elevated in a broad spectrum of cancers and has been consistently associated with tumor metastasis and poor clinical prognosis. Mechanistically, HOTAIR regulates key cellular processes by interacting with multiple molecular partners, thereby promoting tumor cell proliferation, invasion, survival, drug resistance, and metastatic potential in experimental cancer models [256]. 

HOTAIR is an intergenic lncRNA transcribed from the HOXC locus that acts in trans by recruiting chromatin-modifying complexes. One of its best-characterized mechanisms involves the recruitment of the PRC2 to target genomic regions, leading to transcriptional repression of genes within the HOXD cluster and other loci [257].

Importantly, circulating HOTAIR has been detected in the serum and plasma of patients with several malignancies, including breast, colorectal, gastric, and cervical cancers [243]. Elevated circulating HOTAIR levels have been associated with distant metastasis and poor prognosis, particularly in colorectal cancer. These observations are consistent with HOTAIR’s mechanistic role in promoting tumor progression through PRC2-mediated epigenetic reprogramming [258].

#### 4.3.4. Additional Circulating Long Non-Coding RNA Candidates

Several other c-lncRNAs have also demonstrated biomarker potential in specific cancers. Urothelial Cancer Associated 1 (UCA1) is readily detectable in urine and demonstrates strong diagnostic performance for bladder cancer, with a pooled sensitivity of 83% and specificity of 86% across seven clinical studies [244]. Highly Upregulated in Liver Cancer (HULC) is elevated in the plasma of patients with hepatocellular carcinoma and has been proposed as a complementary biomarker to AFP in HCC screening [245,246]. Conversely, tumor-suppressive lncRNAs such as Growth Arrest-Specific 5 (GAS5) and Maternally Expressed Gene 3 (MEG3) are frequently downregulated in the circulation of cancer patients. Circulating GAS5 levels are significantly reduced in multiple malignancies, including NSCLC, breast cancer, colorectal cancer, and pancreatic neoplasms, supporting its potential as a minimally invasive diagnostic biomarker [247]. MEG3 is downregulated across multiple malignancies, including reduced expression in circulating compartments such as plasma, serum, and exosomes in CRC, pancreatic neoplasms, and hematologic cancers, supporting its potential as a minimally invasive biomarker [248,249].

### 4.4. Circulating circRNAs as Cancer Biomarkers

The remarkable stability of circRNAs in biofluids, along with their disease-specific expression patterns, has sparked significant interest in their potential as cancer biomarkers. Their covalently closed structure provides resistance to exonuclease-mediated degradation, making them especially stable in extracellular environments such as plasma, serum, and exosomes. Despite these benefits, the clinical evidence supporting c-circRNAs is still less developed than that for miRNAs and lncRNAs, with most studies remaining in the discovery or early validation phases [259]. Representative examples of circRNAs studied as circulating cancer biomarkers are summarized below.

- Gastric cancer. Among the earliest circRNAs proposed as circulating cancer biomarkers is hsa_circ_0000190, whose plasma levels are significantly lower in patients with gastric cancer. Combined analysis of tissue and plasma samples showed diagnostic potential, with an AUC of 0.775, sensitivity of 71%, and specificity of 75% [260]. Similarly, hsa_circ_0001649 (circSHPRH) has been detected in serum and shows promising diagnostic value in gastric cancer, with expression levels associated with pathological differentiation status [261].
- CRC. Several c-circRNAs have been investigated as potential biomarkers in CRC. Serum levels of hsa_circ_0001649 are reduced in CRC patients compared with healthy individuals [262]. In contrast, hsa_circ_0007534 is significantly elevated in the plasma of CRC patients and is associated with advanced clinical stage, metastatic phenotype, poor tumor differentiation, and unfavorable prognosis [263]. Receiver operating characteristic (ROC) analysis demonstrated moderate diagnostic performance, with an AUC of 0.780, sensitivity of 0.92, and specificity of 0.522 [263].
- Hepatocellular carcinoma (HCC). Plasma levels of hsa_circ_0001445 (circSMARCA5) are significantly reduced in HCC patients compared with individuals with cirrhosis, chronic hepatitis B, or healthy controls. Diagnostic analysis reported an AUC of 0.862, suggesting strong diagnostic performance and potential superiority over AFP for distinguishing early-stage HCC from cirrhosis [264]. Another circRNA candidate, circRNA-100338, has been detected in serum exosomes of HCC patients and is associated with metastatic potential, vascular invasion, and recurrence following hepatectomy [265].
- Lung cancer. High-throughput sequencing studies have identified several c-circRNAs with potential diagnostic and prognostic relevance in NSCLC. Plasma circFARSA levels are significantly elevated in NSCLC patients compared with healthy controls [266]. Additionally, exosomal circ-MEMO1 is increased in the serum of NSCLC patients, and its tumor tissue expression correlates with advanced clinical stage and lymph node metastasis [267].

Overall, these findings indicate that c-circRNAs are a promising new type of liquid biopsy biomarker. Nonetheless, larger multicenter validation studies, better analytical methods, and standardized techniques for detecting circRNAs will be needed before they can be used routinely in clinical settings. Representative examples of circRNA species investigated as circulating cancer biomarkers are summarized in Table 4. It should be noted that differential expression patterns reported in the literature, including those in Table 4, are based on study-specific analytical thresholds and may not be directly comparable across studies.

### 4.5. piRNAs and Other Small ncRNAs as Circulating Biomarkers

Evidence supporting circulating piRNAs (c-piRNAs) as cancer biomarkers is emerging but remains relatively limited compared to miRNAs, lncRNAs, and circRNAs. However, several studies have reported altered c-piRNA profiles in cancer patients. For example, serum levels of piR-651 and piR-823 are significantly lower in patients with gastric cancer than in healthy individuals. Both piRNAs show promising diagnostic performance (AUC = 0.841 and 0.812, respectively), and their combined use further enhances diagnostic accuracy (AUC = 0.860). Notably, piR-823 levels correlate with TNM stage, distant metastasis, and response to chemotherapy, underscoring its potential for early detection as well as for monitoring disease progression and therapeutic response [268].

In addition, a large cohort study involving more than 1000 participants identified serum piR-54265 as an independent diagnostic and prognostic biomarker for CRC. This piRNA demonstrated high diagnostic accuracy (AUC = 0.896) and outperformed conventional biomarkers. Importantly, circulating piR-54265 levels declined markedly following tumor resection, increased again at relapse, and were capable of predicting CRC development up to three years before clinical diagnosis, suggesting potential value for both early detection and longitudinal disease monitoring [269].

Another emerging class ofc-ncRNA biomarkers comprises tsRNAs. These molecules, including tRFs and tiRNAs, are generated through specific cleavage of mature or precursor tRNAs by nucleases such as angiogenin and Dicer [270]. TsRNAs circulate stably in body fluids such as plasma and serum and have recently been described as a “rising star in liquid biopsy” [271]. Several circulating tsRNAs have demonstrated biomarker potential across different cancer types. For example, tRF-Arg-CCT-017 and tiRNA-Phe-GAA-003 have been reported as diagnostic candidates in breast cancer, while AS-tDR-007333 and a three-tsRNA diagnostic signature (AUC = 0.92) have been identified in lung cancer. In colorectal cancer, tRF-35-PNR8YP9LON4VN1 and 5′-tRF-GlyGCC exhibit disease-associated dysregulation and clinically relevant diagnostic or prognostic associations [271]. Given that this class of c-ncRNAs has been thoroughly reviewed elsewhere [271], they will not be discussed further in detail here.

Additional small ncRNA species may also play a role in future liquid biopsy biomarker panels. Small nucleolar RNA–derived fragments (sdRNAs) are an emerging class of regulatory small ncRNAs involved in cancer biology. Although individual sdRNAs have been detected in patient serum, the broader presence of sdRNAs and snRNA-derived fragments in circulating biofluids, and their potential clinical utility, remain largely unexplored [272,273]. As high-throughput sequencing technologies and bioinformatic pipelines continue to advance, the detection and annotation of these ncRNA species are expected to become more accurate, potentially enhancing their role as multi-analyte circulating biomarker panels.

Representative examples of emerging small ncRNA species investigated as circulating cancer biomarkers are summarized in Table 5. Differential expression patterns reported in the literature, including those in Table 5, are based on study-specific analytical thresholds and may not be directly comparable across studies.

### 4.6. Non-Coding RNA Biomarkers Across Biofluid Types

Although blood (serum and plasma) represents the most extensively studied source of c-ncRNAs, these molecules have also been detected in a wide range of additional body fluids, each offering context-specific diagnostic advantages [274,275].

Urine is particularly informative for urogenital tract malignancies. In addition to the established prostate cancer biomarker PCA3, urinary ncRNAs have demonstrated diagnostic potential in several urological cancers [276]. For example, Urinary miR-210 and miR-96 have been proposed as non-invasive biomarkers for bladder cancer detection, supported by studies showing strong diagnostic performance [277]. The lncRNA UCA1 is also highlighted as a regulator associated with bladder cancer biology [278].

Salivary ncRNAs have been primarily investigated in head and neck squamous cell carcinoma (HNSCC) and oral cancers [279]. Several salivary miRNAs, including miR-31, miR-125a, and miR-200a, are differentially expressed in patients with oral squamous cell carcinoma, with reported diagnostic AUC values ranging from 0.65 to 0.82 [280,281]. In addition, salivary transcriptomic profiling has identified candidate miRNA signatures for pancreatic and lung cancer detection, suggesting that salivary biomarkers may reflect both local and systemic disease processes [282].

For central nervous system tumors, cerebrospinal fluid (CSF) provides a tumor-proximal biofluid that is often enriched in tumor-derived nucleic acids [283]. Elevated CSF levels of miR-21 have been consistently reported in glioblastoma patients and correlate with tumor burden and treatment response [284]. Furthermore, exosomal miRNA profiles in CSF have shown greater accuracy in distinguishing glioma subtypes compared to serum-based assays [285].

Malignant pleural effusions contain abundant tumor-derived ncRNAs, with microRNA signatures that closely reflect the molecular phenotype of the primary tumor. In pleural mesothelioma, for example, miRNA panels derived from effusion supernatant achieve excellent diagnostic performance, with AUC values frequently exceeding 0.90 for distinguishing malignant from benign effusions. Similar principles apply to malignant ascites, where tumor shed cells, exosomes, and free nucleic acids accumulate in the peritoneal cavity, generating ncRNA profiles that mirror the biology of the underlying malignancy. Although these fluids are less accessible than blood, they provide a highly enriched, tumor-proximal source of ncRNA biomarkers, particularly in patients with advanced disease, where serous cavity involvement is common [286].

### 4.7. Diagnostic, Prognostic, and Predictive Applications

The clinical utility of c-ncRNA biomarkers spans three principal application domains: early cancer detection, prognostication, and treatment monitoring [28].

- Early detection and screening. The studies reviewed above collectively demonstrate that c-ncRNA signatures can detect cancer in asymptomatic or early-stage individuals, often with diagnostic performance exceeding that of conventional protein biomarkers. One of the most compelling examples is the miR-Test, a 13-miRNA serum signature prospectively validated in the Multicenter Italian Lung Detection (MILD) trial, which demonstrated the ability to identify lung cancer up to two years before CT-based diagnosis [193]. Future screening strategies may incorporate multi-ncRNA panels combining miRNAs, lncRNAs, and circRNAs to capture complementary aspects of tumor biology.
- Prognostication and risk stratification. C-ncRNA levels often correlate with established prognostic indicators, such as tumor stage, histological grade, lymph node involvement, and distant metastases. In various cancer types such as breast, lung, and, prostate and colorectal cancers, specific ncRNA signatures have been identified as independent prognostic factors in multivariate analyses that account for traditional clinicopathological variables, suggesting they provide biologically unique prognostic information [287].
- Treatment monitoring and predictive biomarkers. Dynamic changes in c-ncRNA levels during therapy can serve as pharmacodynamic biomarkers indicating treatment effectiveness. For instance, c-miRNAs such as miR-21 and miR-155 are frequently upregulated in various cancers, including breast, lung, colorectal, gastric, hepatocellular, and pancreatic cancers, and are increasingly used as dynamic biomarkers to track treatment response. Their levels often vary with tumor burden, supporting their usefulness for ongoing response assessment during systemic therapy [23]. Specific ncRNA signatures have been linked to immune-therapy response and resistance, particularly in the context of immune checkpoint inhibitors. Several miRNAs (such as miR-34, miR-138, and miR-155) and lncRNAs (including NEAT1, MALAT1, AFAP1-AS1, and Tim3-lncRNA) modulate PD-1/PD-L1, CTLA-4, and TIM-3 pathways, influencing T-cell exhaustion, immune escape, and resistance to checkpoint blockade [288]. These observations raise the possibility of ncRNA-guided therapeutic stratification in precision oncology.

## 5. Multi-Analyte Biomarker Strategies: Integrating ncRNAs with Complementary Circulating Analytes

Despite the considerable promise of individual ncRNA biomarkers discussed in the preceding sections, single-analyte approaches remain inherently limited in their ability to capture the full biological complexity of cancer. Each molecular class accessible through liquid biopsy, including but not limited to ncRNAs, cfDNA, and circulating proteins, reflects a distinct layer of tumor biology, and no single analyte type can comprehensively represent all clinically relevant aspects of disease. The following subsections provide representative examples illustrating the potential of multi-analyte liquid biopsy strategies rather than an exhaustive catalog of all reported biomarker combinations.

### 5.1. Rationale for Multi-Analyte Liquid Biopsy Approaches

The diagnostic performance of any individual biomarker is constrained by inherent sensitivity–specificity trade-offs, which reflect both biological variability and technical noise associated with measuring a single molecular species [289]. C-miRNAs, for example, provide insight into post-transcriptional regulatory states but may be influenced by non-tumor sources, hemolysis-related artifacts, and physiological variation. CfDNA captures somatic mutations and methylation patterns, yet it is often present at very low concentrations in early-stage disease. Protein biomarkers reflect secretory and proteolytic activity but frequently lack sufficient cancer specificity. Thus, each analyte class offers only a partial and complementary view of tumor biology [36].

The rationale for multi-analyte liquid biopsy is based on both biological complementarity and statistical principles. Since each analyte type, such as ncRNAs, cfDNAs, and proteins, captures different, only partially overlapping aspects of tumor biology, combining features from multiple molecular layers broadens the information available to classification models. This approach reduces the influence of biomarker-specific noise and biological variability, enabling combined panels to achieve greater sensitivity and specificity than any single biomarker. Recent multimodal studies highlighted in this review show significantly improved diagnostic performance and more comprehensive tumor characterization when multiple analytes are analyzed together [18].

This principle has already been validated in several landmark studies. A notable example is the CancerSEEK multi-analyte blood test developed by Cohen et al. [290]. which combined eight circulating protein biomarkers with cfDNA mutation analysis to detect eight common cancer types with a median sensitivity of 70% at a specificity exceeding 99%.

**Figure 3:**
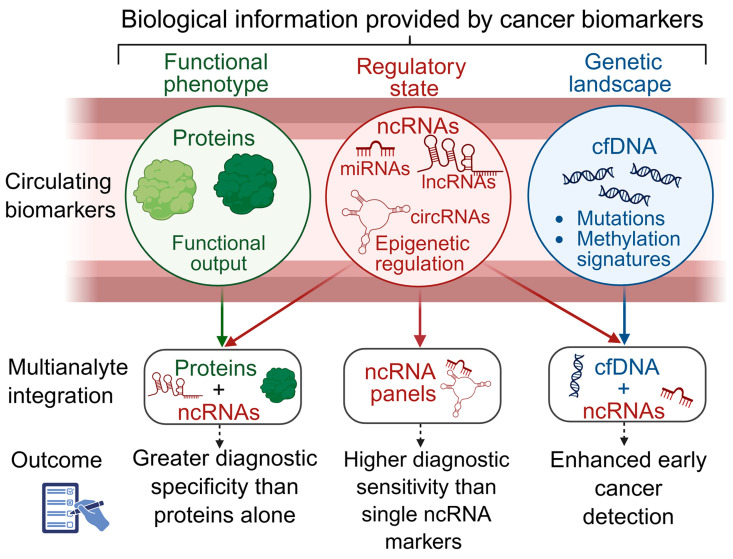
Complementary biological information captured by multi-analyte liquid biopsy biomarkers. Circulating biomarkers provide distinct yet complementary layers of information about tumor biology. Protein biomarkers reflect the tumor’s functional phenotype, including metabolic and secretory changes. Circulating ncRNAs reflect regulatory network activity and epigenetic control of gene expression, while cfDNAs provide insights into the tumor’s genomic landscape, such as somatic mutations and DNA methylation patterns. Combining these different biomarker classes into multi-analyte liquid biopsy panels could improve diagnostic specificity and sensitivity and aid in early cancer detection. This schematic illustrates general principles of biomarkers and does not depict cancer-type or stage-specific changes. Created in BioRender. Papaneophytou, C. (2026) https://BioRender.com/sqo36q1 accessed on 16 March 2026.

Following our recent review [36] highlighting the potential of combining c-miRNAs with cell-free DNA and protein biomarkers in multi-analyte liquid biopsy strategies for various diseases, including cancer diagnosis, prognosis, and disease monitoring, we now expand that framework in several ways directions. First, the ncRNA component is broadened beyond miRNAs to include lncRNAs, circRNAs, and other emerging ncRNA species. Second, we consider integrating multiple ncRNA classes into a single biomarker panel, reflecting the interconnected nature of ncRNA regulatory networks. Third, we examine the potential to integrate ncRNAs with other circulating biomarkers, such as cfDNAs and proteins, to enhance diagnostic and prognostic performance. The examples presented below illustrate the conceptual and clinical potential of multi-analyte liquid biopsy approaches. Rather than providing an exhaustive catalog of all reported biomarker combinations, the discussion focuses on key methodological advances and underlying biological principles. The complementary biological information captured by the major circulating biomarker classes is summarized in Figure 3.

### 5.2. Integration of Multiple Non-Coding RNA Classes

Beyond combining ncRNAs with other circulating analytes, an emerging strategy is the simultaneous profiling of multiple ncRNA species, such as miRNAs, lncRNAs, circRNAs, and other small RNAs, within unified biomarker frameworks. Because these RNA classes regulate gene expression at distinct yet interconnected levels, their joint measurement can capture broader aspects of tumor regulatory states than any single ncRNA class alone. Importantly, cancer-associated RNA networks are inherently multidimensional, and moving beyond single-molecule assays toward integrated, network-level signatures may substantially enhance diagnostic and prognostic performance [33].

Several studies have started to demonstrate the biological and clinical importance of combining multiple ncRNA classes. For example, Tian et al. [291] built an integrated circRNA–miRNA–mRNA (ceRNA) network in Wilms tumor and found that specific circRNAs and their corresponding miRNAs exhibit coordinated but opposite expression patterns that regulate downstream mRNA targets involved in cell-cycle progression and immune-related pathways. These findings suggest that measuring circRNAs and their functionally linked miRNAs can indirectly reflect ceRNA circuit activity, offering insights into regulatory network behaviors that affect tumor traits.

Additional studies examining multiple ncRNA classes within the same tumor type further support this integrative perspective. For instance, analyses of HOTAIR, MIR155HG, TERC, and polymorphisms in miR-155, miR-196a2, and miR-146a in papillary thyroid cancer have demonstrated that distinct ncRNA species can provide complementary regulatory information relevant to cancer susceptibility and prognosis [292]. Similarly, the extensive catalog of HOTAIR–miRNA interactions described by Cantile et al. illustrates how lncRNAs and miRNAs participate in tightly coupled ceRNA circuits regulating processes such as proliferation, epithelial–mesenchymal transition (EMT), immune signaling, and therapy resistance [293]. These multilayered regulatory relationships provide a mechanistic rationale for integrating different ncRNA classes within biomarker panels, as each class captures distinct but complementary aspects of tumor biology.

Mechanistic studies also highlight specific regulatory axes that may serve as clinically informative biomarker combinations. For example, miR-21 interacts with oncogenic lncRNAs such as HOTAIR and MALAT1 across multiple tumor types, including breast, lung, colorectal, gastric, hepatocellular, prostate, and pancreatic cancers, and these regulatory axes have been proposed as promising diagnostic and prognostic markers [294].

The integration of multiple ncRNA classes may also improve the robustness and analytical reliability of biomarker panels by reducing the influence of biological and pre-analytical variability affecting individual molecules. Different ncRNA types exhibit distinct stability profiles in circulation: circRNAs display exceptional stability due to their covalently closed structure, miRNAs are stabilized through AGO2 binding or extracellular vesicle encapsulation, and lncRNAs generally exhibit intermediate stability [295]. Panels incorporating several ncRNA classes may therefore better capture heterogeneous molecular signals derived from tumor cells, stromal components, and the tumor microenvironment while maintaining analytical reliability across variable sample conditions, including differences in sample type, processing time, storage conditions, and RNA integrity.

A clear example of this approach was reported in urinary bladder cancer, where Eissa et al. quantified a multi-class ceRNA network consisting of lncRNA miR-497HG, miR-324-5p, miR-4738-3p, and their mRNA targets (FOSB and RCAN1). This integrated panel captured heterogeneous tumor-derived signals with high analytical robustness and achieved markedly superior discrimination between cancer patients and controls compared with single-analyte assays [296].

More broadly, panels integrating multiple ncRNA classes can exploit the complementary biological properties of each RNA species to capture a wider spectrum of tumor-derived signals. A recent review by Guz et al. [297] highlighted that circRNAs exhibit exceptional biochemical stability and accumulate in extracellular fluids such as plasma, saliva, and urine, making them analytically robust biomarkers. In contrast, miRNAs circulate primarily in AGO protein complexes or extracellular vesicles, reflecting dynamic regulatory responses to tumor-associated processes. Importantly, circRNAs and miRNAs frequently participate in tightly coupled ceRNA regulatory networks, such as circRNA/miR-141/mRNA signaling axes that regulate epithelial–mesenchymal transition, immune evasion, and metastatic progression across multiple cancer types.

Together, these observations illustrate how integrating distinct ncRNA classes within a single biomarker panel may improve the detection of heterogeneous tumor signals while maintaining analytical stability across diverse sample conditions

### 5.3. Non-Coding RNAs Combined with Cell-Free DNA

The integration of c-ncRNAs with cfDNA is among the most actively investigated multi-analyte liquid biopsy strategies. These two analyte classes capture complementary aspects of tumor biology: cfDNA reflects genomic and epigenomic alterations, including somatic mutations, copy number variations, and DNA methylation patterns, whereas ncRNAs provide insight into regulatory network states and post-transcriptional gene expression programs [36,298]. The combined analysis of these molecules therefore offers a more comprehensive representation of tumor molecular dynamics than either analyte alone.

One of the principal motivations for combining cfDNA with c-ncRNA biomarkers is the limited sensitivity of mutation-based cfDNA assays in early-stage cancers. Early tumors often release very small amounts of ctDNA, sometimes fewer than one mutant DNA molecule per milliliter of plasma. As a result, clinically important variants may be below the detection limit of even highly sensitive sequencing technologies. The limitations of ctDNA-only detection were clearly demonstrated by the CancerSEEK study, which found that many early-stage tumors do not shed detectable ctDNA despite optimized barcoding and deep sequencing [290]. By combining a targeted ctDNA mutation panel with eight circulating protein biomarkers, CancerSEEK achieved a median sensitivity of 70% across eight cancer types, with a specificity greater than 99%, detecting about 43% of stage I cancers and thus outperforming mutation-only methods [290]. Importantly, the authors noted that additional circulating analytes, including miRNAs, mRNA transcripts, and methylated DNA, could be incorporated to further enhance diagnostic sensitivity and improve tissue-of-origin prediction.

This rationale is supported by a growing body of evidence demonstrating that circulating RNA (c-RNA) species remain abundant and biologically informative even when ctDNA levels are extremely low. C-RNA profiling can detect mutations, gene fusions, and transcriptional changes that may not be observable through DNA-based assays alone. In addition, ncRNAs such as miRNAs, lncRNAs, circRNAs, and piRNAs, capture dynamic gene regulatory processes associated with tumor growth, immune signaling, and cellular stress responses [142]. Furthermore, several studies have specifically demonstrated the value of integrating signals from circulating RNA and DNA. For example, Ward Gahlawat et al. [299] reported that cfDNA abundance is often insufficient for early cancer detection, whereas c-miRNAs provide robust and biologically meaningful signals. The authors therefore proposed rational multi-analyte strategies combining cfDNA mutation analysis with c-miRNA profiling to improve both sensitivity and specificity in liquid biopsy diagnostics.

Integrated DNA–RNA profiling has also shown clinical utility in cancer prognosis and treatment monitoring. In prostate cancer, Fettke et al. [300] developed a single-tube multi-analyte liquid biopsy combining cfDNA and cfRNA analysis of the androgen receptor (AR). This approach enabled the simultaneous detection of AR copy-number gain, AR mutations, and splice variants (AR-V7 and AR-V9). Patients with combined AR genomic and transcriptomic alterations showed significantly worse PSA progression-free survival, clinical progression-free survival, and overall survival. These results were independently confirmed in an external cohort, supporting the clinical importance of integrated cfDNA–cfRNA analysis for risk assessment in metastatic castration-resistant prostate cancer (mCRPC).

Beyond mutation analysis, cfDNA methylation profiling has emerged as a promising strategy for cancer detection because tumor-specific methylation patterns are often more consistent across cancer types than somatic mutations [301]. Combining cfDNA methylation markers with c-ncRNAs enables simultaneous collection of epigenomic and transcriptomic regulatory data from a single liquid biopsy. Since epigenetic changes often precede transcriptional dysregulation, measuring DNA methylation alongside ncRNA expression may provide complementary insights into the same regulatory disruptions in tumor cells.

Taken together, these studies demonstrate how combining cfDNA-based genomic and epigenomic signals with c-ncRNA biomarkers can significantly enhance the sensitivity and biological interpretability of liquid biopsy tests, especially in early-stage disease, where single-analyte approaches often encounter fundamental detection limitations.

### 5.4. Non-Coding RNAs Combined with Protein Biomarkers

The integration of c-ncRNAs with established protein biomarkers addresses a well-recognized limitation of conventional cancer diagnostics: most protein markers lack sufficient specificity for stand-alone diagnosis, and their elevation in benign conditions often leads to unacceptable false-positive rates [28]. Combining protein biomarkers with ncRNA signatures, therefore, represents a promising strategy to improve diagnostic accuracy in liquid biopsy applications.

Prostate cancer provides one of the clearest examples of this approach. As discussed above, PSA screening achieves high sensitivity for prostate cancer detection but is limited by poor specificity, leading to substantial overdiagnosis and unnecessary biopsies. Kachakova et al. [302] demonstrated that integrating c-miRNAs with PSA improves diagnostic performance. In their study of prostate cancer patients and matched controls, plasma levels of let-7c, miR-30c, miR-141, and miR-375 were measured, along with serum PSA. While miR-375 alone outperformed PSA (AUC 0.809 vs. 0.710), the strongest diagnostic performance was achieved through multi-analyte integration: combinations of miRNAs with PSA reached AUC values up to 0.877, with sensitivity and specificity exceeding 85% and 80%, respectively. These findings demonstrate how c-ncRNAs can provide complementary biological information, thereby improving the diagnostic accuracy of traditional protein markers.

As discussed above, an established clinical example of multi-analyte integration is the combination of PSA with the urinary lncRNA PCA3 score, which is currently approved by the U.S. FDA to guide repeat biopsy decisions in prostate cancer patients. The combined assessment of PSA and PCA3 enhances diagnostic specificity compared to either marker alone and represents one of the earliest successful implementations of ncRNA-based multi-analyte testing in clinical practice [303].

Emerging evidence also suggests that c-lncRNAs can complement established protein biomarkers in several cancer types. For example, Zhang et al. [304] reported that circulating HOTAIR is significantly elevated in both breast cancer tissues and plasma. HOTAIR demonstrated superior diagnostic performance compared with the traditional serum markers CEA and CA15-3 (AUCs of 0.80, 0.50, and 0.65, respectively). Importantly, combining HOTAIR with CEA and CA15-3 further improved diagnostic accuracy (AUC 0.82), illustrating that ncRNAs may enhance existing biomarker panels rather than replace them. In addition, circulating HOTAIR levels decreased following surgical tumor removal and correlated with tumor tissue expression, suggesting potential utility for treatment monitoring.

In lung cancer, Yuan et al. [305] identified a circulating four-lncRNA plasma panel (RMRP, NEAT1, TUG1, and MALAT1) that demonstrated strong diagnostic performance for non-small-cell lung cancer (NSCLC), with AUC values of 0.86 in the training cohort and 0.89 in an independent validation cohort. The panel maintained high sensitivity even in early-stage disease. When compared with conventional serum protein markers (CEA, CA125, CYFRA21-1, SCC, and NSE), the lncRNA panel showed higher sensitivity, particularly in adenocarcinoma. Integrating the lncRNA panel with protein biomarkers further improved diagnostic performance, reaching AUC values of 0.85 in adenocarcinoma and 0.93 in squamous cell carcinoma.

Comparable findings have been reported in colorectal cancer. Wang et al. [306] identified a three-lncRNA serum signature (LOC285194, RP11-462C24.1, and Nbla12061) that distinguished colorectal cancer patients from healthy individuals with higher accuracy than conventional protein markers such as CEA, CA19-9, CA125, and CA724. While the lncRNA panel alone achieved an AUC of 0.793, combining the lncRNA signature with protein biomarkers further increased diagnostic accuracy (AUC 0.845–0.855), again highlighting the complementary value of multi-analyte approaches.

More broadly, the principle of integrating orthogonal biomarker classes has been validated by the CancerSEEK study, which demonstrated that combining circulating proteins with tumor-derived genetic alterations substantially improves multi-cancer detection performance [307]. Although the original CancerSEEK panel did not include ncRNAs, the authors noted that adding more analyte classes, including microRNAs, could further improve diagnostic sensitivity and tissue-of-origin prediction. Early discovery-stage studies applying this idea have already begun testing combined panels of c-miRNAs alongside established protein markers such as CEA, CA19-9, AFP, and CYFRA21-1 across different cancer types, including colorectal, pancreatic, gastric, hepatocellular, and lung cancers. While many of these studies show promising diagnostic accuracy, most are still in the training-cohort phase and require independent prospective validation before they can be used in clinical settings.

## 6. Technical and Clinical Challenges in ncRNA Liquid Biopsy

Despite significant progress in identifying c-ncRNAs as cancer biomarkers, numerous technical, analytical, and clinical hurdles still hinder their routine use in clinical practice. These challenges affect the entire analytical process, from pre-analytical sample handling and ncRNA detection methods to data analysis and large-scale clinical validation. Overcoming these obstacles is crucial for moving ncRNA-based liquid biopsy tests from research to standard clinical diagnostics.

Additionally, multi-analyte liquid biopsy strategies that combine c-miRNAs, cfDNA, and protein biomarkers introduce further analytical and methodological challenges. Several of these issues have been discussed extensively elsewhere and are summarized briefly below [177,308,309,310].

### 6.1. Pre-Analytical Variables and Sample Processing

One of the main sources of variability inc-ncRNA analysis comes from pre-analytical factors, including how samples are collected, processed, and stored. Differences in blood collection tubes, anticoagulants, centrifugation methods, and storage procedures can significantly affect measured ncRNA levels. For instance, hemolysis during blood collection can release large amounts of intracellular miRNAs, such as miR-16 and miR-451, from red blood cells, potentially interfering with biomarker measurements. Likewise, delays in plasma separation after blood collection can change ncRNA profiles due to ongoing cellular breakdown and RNA release. The choice between serum and plasma also creates variability, since clotting during serum preparation can release additional RNAs from platelets and white blood cells. Additionally, repeated freeze–thaw cycles may damage RNA and increase measurement variability. Therefore, standardized protocols for sample collection, processing, and storage are essential to ensure consistent results across studies and laboratories.

### 6.2. Analytical and Detection Methodologies

The detection and measurement of c-ncRNAs depend on various analytical platforms, including RT-qPCR, microarrays, and NGS. Each method offers unique benefits and drawbacks in terms of sensitivity, dynamic range, and throughput.

RT-qPCR remains the most commonly used method for targeted ncRNA quantification because of its high sensitivity, specificity, and relatively low cost. However, this method requires prior knowledge of candidate biomarkers and can introduce variability during reverse transcription and amplification. Microarray-based platforms allow for the simultaneous analysis of many RNA molecules but generally have lower sensitivity than sequencing-based methods. Conversely, NGS offers comprehensive and unbiased profiling of c-RNA populations, enabling the discovery of new ncRNA species and isoforms. Still, sequencing approaches tend to be more costly and computationally intensive. Additionally, variations in RNA isolation techniques and library preparation protocols may cause systematic biases, making cross-study comparisons more difficult. The lack of standardized analytical pipelines, therefore, remains a major barrier to the consistent detection of c-ncRNA biomarkers.

### 6.3. Normalization and Data Interpretation

Accurate normalization is one of the most challenging aspects of quantifying c-ncRNAs. Unlike intracellular RNA analysis, where housekeeping genes can often be used as stable references, finding reliable endogenous normalization controls in biofluids has been difficult. Many commonly used reference miRNAs show variable expression across individuals or disease states. Alternative strategies, such as synthetic spike-in controls, global mean normalization, and panels of reference miRNAs, have been suggested; however, none have yet become a universally accepted standard. Variability in normalization methods across studies leads to inconsistent results and makes it hard to compare reported biomarker signatures. Developing robust normalization frameworks will be crucial for translating ncRNA-based tests into clinical use.

### 6.4. Biological Complexity and Tumor Heterogeneity

Another major challenge comes from the biological complexity of c- ncRNAs. Tumor-derived ncRNAs make up only a small part of the total c-RNA, which also includes RNAs from normal tissues, immune cells, and other sources. Additionally, c-ncRNAs can be produced through various mechanisms, including passive release from dying tumor cells, active secretion via extracellular vesicles, or binding to RNA-binding proteins like Argonaute complexes.

Tumor heterogeneity adds further complexity to biomarker development. Genetic and epigenetic differences among tumor subclones, along with variations between primary tumors and metastatic lesions, can result in diverse ncRNA expression patterns. These factors may affect both the sensitivity and specificity of c-ncRNA biomarkers, underscoring the potential benefits of multi-analyte strategies that target distinct aspects of tumor biology.

### 6.5. Clinical Validation and Standardization

Perhaps the most important obstacle to clinical implementation is the scarcity of large, prospective validation studies. Many ncRNA biomarker candidates have been discovered in relatively small case–control cohorts, which might be prone to selection bias and overfitting. Therefore, independent validation in large, multicenter cohorts is essential to verify the reproducibility and clinical usefulness of proposed biomarker panels.

Regulatory approval for ncRNA-based diagnostic assays also demands thorough analytical validation, evidence of clinical benefit, and standardized testing procedures. While the approval of the urinary lncRNA PCA3 assay for prostate cancer marks a significant milestone, similar examples remain few. Future advancements will rely on coordinated efforts to standardize methods, develop reference materials, and perform prospective clinical trials to evaluate ncRNA-based liquid biopsy tests.

## 7. Challenges, Future Directions, and Clinical Translation

Despite the substantial body of evidence reviewed in the previous sections, translating c-ncRNA biomarkers from research findings into routine clinical tools remains a persistent challenge. Although many studies have demonstrated the potential of ncRNAs as diagnostic and prognostic biomarkers, their transition from experimental validation to widespread clinical use faces numerous scientific, technical, and regulatory hurdles. Identifying reliable ncRNA biomarkers is inherently complex.

Establishing clinically meaningful threshold values requires large, well-characterized patient cohorts, while biological variables such as age, sex, and population heterogeneity must also be considered. Moreover, relatively few single biomarkers exhibit sufficient disease specificity, as many ncRNAs display overlapping expression patterns across multiple pathological conditions [178]. Notably, introducing new biomarkers into clinical practice involves navigating regulatory approvals, such as those required by the U.S. FDA or European Medicines Agency (EMA), which generally demand rigorous evaluation of biomarker safety and effectiveness before clinical use. Additionally, ethical considerations must be addressed, including obtaining informed patient consent, safeguarding patient privacy, and establishing secure data management protocols to prevent misuse or unauthorized access [311].

### 7.1. Standardization and Pre-Analytical Harmonization

Pre-analytical variability and normalization challenges remain some of the biggest barriers to clinical adoption. Although these issues are well recognized, progress in resolving them has been slow. Several key developments will be needed moving forward. International initiatives, including the Extracellular RNA Communication Consortium (ERCC) [312] have begun establishing standardized operating procedures for c-RNA analysis. However, broader implementation and adherence to these protocols across clinical laboratories are required to ensure reproducibility and comparability of results [313]. The development of certified reference materials, analogous to those used in clinical chemistry, would provide the metrological traceability needed for inter-laboratory calibration and proficiency testing of ncRNA assays, improving reproducibility and comparability across laboratories [314]. In addition, standardized reporting guidelines analogous to the MIQE framework should be extended specifically to c-ncRNA studies to improve transparency, reproducibility, and cross-study comparison [315].

### 7.2. Clinical Validation and Regulatory Pathways

Although c-ncRNAs show significant potential for various clinical applications, such as predicting treatment response, only a few clinical trials have been launched to date. A recent review by Chang et al. [94] highlights that most c-ncRNA biomarkers have been identified in small, retrospective case–control studies with heterogeneous designs and limited clinical generalizability. Meaningful clinical translation will therefore require large-scale, prospective, multicenter validation studies conducted in realistic populations, including individuals with benign conditions, early-stage disease, and treatment-modified molecular profiles.

A search of ClinicalTrials.gov (https://clinicaltrials.gov/, accessed on 14 March 2026) identified 48 cancer-related clinical studies that explicitly evaluate c-miRNAs. Among these, 15 studies have been completed, while the remainder are recruiting, active, or of unknown status. Notably, no registered clinical trials were identified that evaluate c-lncRNAs or c-circRNAs as dynamic biomarkers. This imbalance highlights that, despite growing interest in non-coding RNA biomarkers, only c-miRNAs have advanced to meaningful clinical trials.

Future progress will require prospectively designed studies where therapeutic decisions are guided by longitudinal changes in ncRNA profiles, moving beyond retrospective observational analyses. Such approaches may be especially relevant in immunotherapy, where dynamic molecular monitoring could help predict treatment responses and inform therapeutic adjustments.

Regulatory pathways for ncRNA-based diagnostics are still largely undefined, aside from the example set by the urinary PCA3 assay for prostate cancer. Initiating early engagement with regulatory agencies during biomarker development, especially as biomarkers move from verification to clinical validation, will be crucial for establishing evidentiary standards for approval. Companion diagnostic strategies, in which ncRNA biomarker panels guide specific treatments, might offer a particularly promising regulatory pathway, as they directly link biomarker approval to proven clinical utility.

### 7.3. Single-Cell and Spatial Technologies for ncRNA Biomarker Refinement

Single-cell RNA sequencing and spatial transcriptomic technologies are rapidly transforming the resolution at which ncRNA expression can be studied within tumor ecosystems. These approaches offer two major opportunities for improving ncRNA biomarker development [316,317].

- First, single-cell analysis can help identify the cellular origins of c-ncRNAs, distinguishing tumor-derived molecules from those released by stromal or immune cells. This information may enable the development of more tumor-specific circulating biomarker panels.
- Second, spatial transcriptomic approaches can reveal microenvironment-dependent regulatory programs that influence ncRNA expression and secretion. Mapping ncRNA expression within tumor tissue architecture may therefore help identify molecules more likely to be released into circulation and detectable through liquid biopsy.

Integrating single-cell and spatial transcriptomic data with matched liquid biopsy profiles from the same patients is an important, largely unexplored research area that could significantly enhance the biological understanding and clinical utility of c-ncRNA biomarkers.

### 7.4. Attractor-State Modeling and Network-Informed Biomarker Design

The attractor-state framework outlined in Section 3 offers a conceptual foundation for a markedly different approach to designing biomarker panels. Since ncRNAs function within interconnected regulatory networks and influence multiple oncogenic pathways, biomarker panels that aim to capture these coordinated network states, rather than relying solely on differential expression, may better represent the tumor’s underlying molecular phenotype [318].

Computational methods for inferring attractor states from bulk and single-cell transcriptomic data are advancing rapidly. Applying these approaches to c-ncRNA profiles may enable the development of “network-state biomarkers” that classify tumors according to their position within the regulatory landscape rather than relying on individual molecular markers [319].

Such strategies may be inherently more robust to molecular heterogeneity because they focus on emergent system-level properties rather than individual components. Realizing this potential will require closer collaboration between the regulatory network modeling and liquid biopsy research communities, which have thus far developed largely independently.

### 7.5. Artificial Intelligence and Integrative Computational Frameworks

Artificial intelligence (AI) and machine learning (ML) techniques are expected to play an increasingly significant role in analyzing and interpreting complex multi-analyte biomarker datasets. Future computational frameworks will need to incorporate several additional capabilities [320].

First, longitudinal liquid biopsy datasets, collected through serial sampling during disease progression and treatment, must be incorporated to track dynamic changes in ncRNA profiles over time. Second, predictive models should integrate molecular features with clinical metadata, including imaging findings, histopathology, and treatment history, to support more comprehensive, clinically relevant decision-making. Finally, developing explainable AI methods will be vital for clinical adoption. Interpretable models that offer biologically meaningful rationales for their predictions will promote both clinician trust and regulatory approval. Emerging approaches like foundation models and transfer learning are beginning to be applied to RNA expression datasets and may enable the creation of robust ncRNA biomarker classifiers that apply across cancer types, patient populations, and analytical method platforms.

### 7.6. Toward Clinical Implementation

Taken together, advances in analytical standardization, prospective clinical validation, single-cell and spatial transcriptomics, network-based biomarker design, and AI-driven integrative analytics outline a plausible pathway toward the clinical implementation of ncRNA-based liquid biopsy approaches.

In the near term, the most realistic scenario involves integrating validated ncRNA panels into multi-analyte liquid biopsy platforms alongside cfDNA and protein biomarkers. These combined strategies could support several clinically relevant applications, including early cancer detection in high-risk populations, monitoring minimal residual disease after curative-intent therapy, and evaluating treatment response during systemic therapy [36]. A practical example demonstrating the value of combining multiple biomarkers comes from prostate cancer diagnostics. Although the lncRNA PCA3 has been approved as a urinary biomarker for prostate cancer, it has limited sensitivity in patients with low-grade disease. Studies have shown that overexpression of the ERG oncogene in prostate cancer is often driven by its fusion with the TMPRSS2 gene [321]. The combined analysis of PCA3 and the TMPRSS2–ERG gene fusion has therefore been shown to enhance diagnostic accuracy over using either marker alone [322].

Achieving these goals will require ongoing interdisciplinary collaboration among molecular biologists, clinical oncologists, bioinformaticians, and regulatory scientists. Together, these advances underscore that ncRNAs are emerging not just as circulating biomarkers but also as system-level indicators of the regulatory landscapes that influence tumor behavior.

## 8. Conclusions

This review has explored the expanding landscape of non-coding RNAs in cancer across two interconnected areas: regulatory network biology and liquid biopsy biomarker development. At the biological level, ncRNAs are key components of gene regulatory networks that control cellular homeostasis, and their dysregulation in cancer contributes to the establishment and maintenance of abnormal phenotypic states. The attractor-state framework offers a system-level view on how disruptions in ncRNA networks can push cells toward stable cancerous states, thereby connecting molecular regulatory mechanisms to cancer phenotypes.

At the diagnostic level, c-ncRNAs, such as miRNAs, lncRNAs, circRNAs, and other small RNA molecules, have shown significant promise as minimally invasive biomarkers for cancer detection, prognosis, and treatment monitoring. Their notable stability in biofluids, combined with the development of increasingly sensitive analytical platforms, supports their potential inclusion in clinical workflows. The use of multiple ncRNA types within combined biomarker panels, along with integrating ncRNAs with complementary circulating analytes like cell-free DNA and protein biomarkers, is a particularly promising approach to enhance diagnostic accuracy beyond what single-analyte methods can provide.

Despite these advances, several obstacles still hinder the routine clinical use of ncRNA-based liquid biopsies. Variability before testing, the lack of universally accepted normalization standards, and the small number of large prospective validation studies continue to limit clinical implementation. Overcoming these barriers will require coordinated interdisciplinary efforts to standardize analytical procedures, develop reference materials, and perform comprehensive multicenter validation studies.

Looking ahead, the convergence of emerging technologies, such as single-cell and spatial transcriptomics, AI-driven biomarker discovery, and network-based biomarker panel design, offers new opportunities to improve understanding and clinical use of c-ncRNA biomarkers. These advances suggest a feasible path toward integrating ncRNA-based liquid biopsy methods into precision oncology, where they could help detect cancer earlier, enhance patient stratification, and enable more flexible monitoring of disease progression and treatment response.

## Figures and Tables

**Table 1:** Major classes of regulatory ^1^ non-coding RNAs: biogenesis, functions, and relevance in cancer ^2^.

ncRNA Class, Length (nt)	Biogenesis	Major Molecular Functions	Relevance in Cancer
microRNAs (miRNAs) ~19–25	RNA polymerase II transcribes pri-miRNAs, processed by Drosha (nucleus) and Dicer (cytoplasm) to produce mature miRNAs incorporated into RISC	Post-transcriptional regulation of gene expression through sequence-specific mRNA degradation or translational repression	Dysregulated miRNA expression promotes oncogenic signaling and impairs tumor suppressor function. Potential biomarkers in liquid biopsies
Long non-coding RNAs (lncRNAs), >200	Transcribed by RNA polymerase II and processed like mRNAs; originating from intergenic, intronic, antisense, or enhancer regions	Regulation of transcriptional programs, chromatin remodeling, epigenetic modification, RNA–RNA interactions, and modulation of protein activity	Involved in tumor initiation, progression, metastasis, and therapy resistance Cancer-type- or context-specific expression patterns
Circular RNAs (circRNAs), variable (hundreds to thousands)	Generated through back-splicing events that covalently link downstream splice donors to upstream splice acceptors	Can sequester miRNAs, regulate transcription and alternative splicing, interact with RNA-binding proteins, and have context-dependent translational potential	High stability and detectability in biofluids, increasingly explored as diagnostic and prognostic biomarkers
Piwi-interacting RNAs (piRNAs), ~24–31	Processed from long single-stranded precursors via PIWI protein-associated pathways independent of Dicer	Transposon silencing, epigenetic regulation, and maintenance of genomic integrity	Dysregulated piRNA expression has been associated with tumorigenesis and cancer progression
Small nucleolar RNAs (snoRNAs), ~60–300	Processed from intronic regions of host genes and primarily localized to the nucleolus	Guide RNA modification; regulates alternative splicing and mRNA processing; may generate snoRNA-derived RNAs	Emerging evidence implicates snoRNAs in altered ribosome biogenesis, cancer metabolism, and tumor progression
Small nuclear RNAs (snRNAs), ~100–300	Transcribed by RNA polymerase II or III and assembled with proteins to form small nuclear ribonucleoproteins	Core components of the spliceosome required for pre-mRNA splicing	Dysregulation of snRNAs and splicing machinery contributes to aberrant transcript processing in cancer
tRNA-derived small RNAs (tsRNAs), ~14–40	Cleavage of precursor or mature tRNAs; includes tRNA-derived fragments and tRNA halves	Regulation of translation, RNA stability, and cellular stress responses	Modulators of tumor growth, metastasis, and therapeutic

^1^ The table highlights the main regulatory ncRNA classes discussed in this review and offers a general classification framework that complements Figure 1; ^2^ Data obtained from [46,47,48].

**Table 2:** Circulating miRNAs as cancer biomarkers across tumor types.

miRNA(s)	Cancer Type	Sample Type	Clinical Application	Performance	Ref.
miR-21, miR-126, miR-210, miR-486-5p	Non-small-cell lung cancer (NSCLC)	Plasma	Early detection	Sensitivity 73–87% Specificity 83–97%	[192]
13-miRNA panel (miR-Test)	Lung cancer	Serum	Prospective screening	Detected lung cancer up to 2 years before CT ^1^-based1 diagnosis	[193]
miR-21, miR-155, miR-10b, miR-145	Breast cancer	Plasma/ Serum	Diagnosis	Pooled AUC ^2^ > 0.85	[194,195]
miR-92a	Colorectal cancer (CRC)	Plasma	Diagnosis	AUC = 0.89	[196]
miR-23a-3p, miR-27a-3p, miR-142-5p, miR-376c-3p	CRC	Serum	Diagnosis	AUC of 0.917 Sensitivity 89% Specificity 81%	[197]
miR-141, miR-375, miR-1290	Prostate cancer	Plasma/ Exosomes	Prognosis	Independent predictors of overall survival	[198,199]

^1^ CT: Computed Tomography; ^2^ AUC: Area Under Curve.

**Table 3:** Representative circulating lncRNAs investigated as cancer biomarkers in liquid biopsy studies ^1^.

lncRNA	Cancer Type	Biofluid	Clinical Application	Key Findings	Ref.
PCA3	Prostate	Urine	Diagnostic	An FDA ^2^-approved biomarker used with PSA ^3^ to guide repeat biopsy decisions	[240]
MALAT1	NSCLC ^4^, breast, gastric	Plasma/ serum	Prognostic	Elevated circulating levels are linked to advanced tumor stage and poorer survival	[241,242]
HOTAIR	Breast, CRC ^5^, gastric	Plasma/ serum	Prognostic	Associated with metastasis and poor clinical outcomes	[243]
UCA1	Bladder cancer	Urine	Diagnostic	Sensitivity and specificity > 80% for bladder cancer detection	[244]
HULC	Hepatocellular carcinoma	Plasma	Diagnostic	Elevated circulating levels are proposed as a complement to AFP ^6^ screening	[245,246]
GAS5	Breast, CRC, pancreatic neoplasms	Plasma/ serum	Tumor- suppressive biomarker	Reduced circulating levels associated with tumor presence	[247]
MEG3	CRC, pancreatic neoplasms, hematologic cancers	Plasma/Serum/Exosomes	Tumor-suppressive biomarker	Downregulated in cancer patients compared with controls	[248,249]

^1^ Beyond their altered circulating levels, these lncRNAs modulate gene expression via chromatin remodeling (HOTAIR, MEG3), transcriptional and splicing regulation (MALAT1, PCA3), or ceRNA-mediated de-repression of oncogenic mRNAs (UCA1, HULC). Tumor-suppressive lncRNAs such as GAS5 and MEG3 normally restrain pro-survival or proliferative gene expression, and their downregulation facilitates malignant progression. Mechanistic descriptions are based on the representative references listed in the table; ^2^ FDA: US Food and Drug Administration; ^3^ PSA: Prostate-specific antigen; ^4^ NSCLC: Non-small-cell lung cancer; ^5^ CRC: Colorectal cancer; ^6^ AFP: Alpha-fetoprotein.

**Table 4:** Representative circulating circRNAs investigated as cancer biomarkers in liquid biopsy studies.

circRNA	Cancer	Biofluid	Expression Pattern ^1^	Clinical Relevance	Key Findings	Ref.
hsa_circ_0000190	Gastric	Plasma	↓ Downregulated	Diagnostic	AUC 0.775, sensitivity 71%, specificity 75%	[260]
hsa_circ_0001649	Gastric/ CRC	Serum	↓ Downregulated	Diagnostic biomarker	Associated with pathological differentiation	[261]
hsa_circ_0001649	CRC	Serum	↓ Downregulated	Diagnostic	Reduced circulating levels in CRC patients	[262]
hsa_circ_0007534	Colorectal	Plasma	↑ Upregulated	Diagnostic/ prognostic	Associated with metastasis and poor differentiation; AUC 0.780	[263]
hsa_circ_0001445 (circSMARCA5)	HCC	Plasma	↓ Downregulated	Diagnostic	AUC 0.862; potential superiority to AFP for early HCC detection	[264]
circRNA-100338	HCC	Serum exosomes	↑ Upregulated	Prognostic	Associated with vascular invasion and post-hepatectomy recurrence	[265]
circFARSA	NSCLC	Plasma	↑ Upregulated	Diagnostic	Elevated levels in NSCLC patients	[266]
circ-MEMO1	NSCLC	Serum exosomes	↑ Upregulated	Prognostic	Correlates with advanced stage and lymph node metastasis	[267]

^1^ Expression patterns indicating upregulation or downregulation reflect the direction of change reported in the original studies; thresholds and statistical criteria vary across studies and were not standardized in this review. Abbreviations: AUC, area under the curve; AFP, alpha-fetoprotein; CRC, colorectal cancer; HCC, hepatocellular carcinoma; NSCLC, non-small-cell lung cancer.

**Table 5:** Emerging small ncRNAs investigated as circulating cancer biomarkers.

ncRNA (Class)	Cancer Type	Biofluid	Expression Pattern ^1^	Clinical Relevance	Key Findings	Ref.
piR-651 (piRNA)	Gastric	Serum	↓ Downregulated	Diagnostic	AUC = 0.841 for distinguishing gastric cancer from controls	[268]
piR-823 (piRNA)	Gastric	Serum	↓ Downregulated	Diagnostic/monitoring	Correlates with TNM stage, metastasis, and chemotherapy response	[268]
piR-54265 (piRNA)	Colorectal	Serum	↑ Upregulated	Diagnostic/prognostic	AUC = 0.896; predicts CRC up to 3 years before diagnosis; levels decline post-surgery and increase at relapse	[269]
tRF-Arg-CCT-017 (tsRNA/tRF)	Breast	Plasma	Dysregulated	Diagnostic	Differential expression in breast cancer patients	[271]
tiRNA-Phe-GAA-003 (tsRNA/tiRNA)	Breast	Plasma	Dysregulated	Diagnostic	Associated with breast cancer detection	[271]
AS-tDR-007333 (tsRNA)	Lung	Plasma	Dysregulated	Diagnostic	Part of a multi-tsRNA diagnostic signature (AUC ≈ 0.92)	[271]
tRF-35-PNR8YP9LON4VN1 (tsRNA/tRF)	Colorectal	Plasma	Dysregulated	Diagnostic/prognostic	Associated with CRC development	[271]
5′-tRF-GlyGCC (tsRNA/tRF)	Colorectal	Plasma	Dysregulated	Diagnostic	Differentially expressed in CRC patients	[271]

^1^ Expression patterns indicating upregulation or downregulation reflect the direction of change reported in the original studies; thresholds and statistical criteria vary across studies and were not standardized in this review. Abbreviations: AUC, area under the curve; TMN, tumor node metastasis; CRC, colorectal cancer

## Data Availability

No new data were created or analyzed in this study. Data sharing is not applicable to this article.
